# State-of-the-Art Development in Liquid Crystal Biochemical Sensors

**DOI:** 10.3390/bios12080577

**Published:** 2022-07-29

**Authors:** Xiyun Zhan, Yanjun Liu, Kun-Lin Yang, Dan Luo

**Affiliations:** 1 Department of Electrical and Electronic Engineering, Southern University of Science and Technology, Xueyuan Road 1088, Shenzhen 518055, China; 11955008@mail.sustech.edu.cn (X.Z.); yjliu@sustech.edu.cn (Y.L.); 2 Department of Chemical and Biomolecular Engineering, National University of Singapore, 4 Engineering Drive 4, Singapore 117576, Singapore

**Keywords:** liquid crystal, stimuli-responsive material, biological analytes, chemical analytes, machine learning, biochemical sensor, label free, high sensitivity

## Abstract

As an emerging stimuli-responsive material, liquid crystal (LC) has attracted great attentions beyond display applications, especially in the area of biochemical sensors. Its high sensitivity and fast response to various biological or chemical analytes make it possible to fabricate a simple, real-time, label-free, and cost-effective LC-based detection platform. Advancements have been achieved in the development of LC-based sensors, both in fundamental research and practical applications. This paper briefly reviews the state-of-the-art research on LC sensors in the biochemical field, from basic properties of LC material to the detection mechanisms of LC sensors that are categorized into LC-solid, LC–aqueous, and LC droplet platforms. In addition, various analytes detected by LCs are presented as a proof of the application value, including metal ions, nucleic acids, proteins, glucose, and some toxic chemical substances. Furthermore, a machine-learning-assisted LC sensing platform is realized to provide a foundation for device intelligence and automatization. It is believed that a portable, convenient, and user-friendly LC-based biochemical sensing device will be achieved in the future.

## 1. Introduction

Liquid crystal (LC), an intermediate state between liquid and solid, was first observed by Reinitzer in 1888 [1]. In general, the phase transition of thermotropic LC occurs within a certain temperature range, from crystalline phase, smectic phase, nematic phase to isotropic phase. It combines the fluidity of liquid with the anisotropic property of crystal and possesses long-range orientational orders. The rod-shaped LC molecules tend to orient parallel to each other along the same direction, represented by director ***n***. The unique intrinsic property of LC makes it extremely sensitive to various external stimuli, including temperature, light, electric field, magnetic field, and chemical and biological molecules, leading to a phase change or orientational change in LC molecules [2,3,4,5,6]. As a stimuli-responsive soft material, LC has been widely exploited in many applications, such as electronic displays, optical imaging, smart windows, soft actuators, and so on [7,8,9]. In addition, LC has recently become a promising candidate in the sensing field.

Since it was first demonstrated by Abbott’s group that LC can be used as an optical signal amplifier to report specific binding events between ligand and receptor in 1998, the development of research on LC sensors has progressed [10]. In the early-stage development of LC sensing systems, they studied the influence of biomolecular interactions on the orientation of LC, which was supported on a solid substrate [11,12,13]. The nanostructured surface was formed by self-assembled monolayers (SAMs) of organosulfur compounds on obliquely deposited gold films. Terminal groups of the SAMs were also decorated with ligands or antibodies. Subsequently, binding events between protein and ligand or protein and antibody were transduced through LC supported on the solid substrate into optical signals. This study provided a universal platform for a more sophisticated design of functionalized substrate to detect a broad spectrum of targets. During the 2000s, researchers also investigated a lot of interfacial biological events between the aqueous phase and the LC phase, including amphiphile adsorption, phospholipid assembly, protein–receptor interaction, enzymatic activity of oligopeptide amphiphiles, and so on [14,15,16,17,18,19]. Brake and co-authors contributed to the exploration of amphiphile and lipid assemblies at the LC interface, as well as the protein binding events at the lipid-decorated surface. These molecular events can be observed through the spatially patterned orientations of LC with optical images. These early findings guided the development of the label-free LC sensing platform without complicated instrumentation.

Over the past decade, more and more types of LC-based sensors have been designed to detect different analytes, including gas molecules, metal ions, nucleic acids, proteins, bacteria, and so on. LC molecules show rapid response to the presence of small analytes or specific binding interactions, then amplify and convert the microscopic molecular signal into a macroscopic optical signal. It can be easily read out by the naked eye under a polarized optical microscope (POM) or collected through optical spectrum. Conventional biochemical sensing technologies usually require time-consuming sample treatments, complicated operation processes, and large-scale and expensive equipment. In contrast, LC-based sensors offer a simple label-free method, with good sensitivity, selectivity, low cost, and rapid response for the detection of biological and chemical species. Hence, LC shows great potential for toxicant detection, environmental monitoring, and disease diagnosis. To date, a number of groups have reviewed the general components and applications related to LC-based biosensors [20,21,22,23]. There are still some missing parts that are worth discussing, such as LC sensing of chemical compounds and machine-learning-assisted LC sensing technology.

This review paper aims to provide a general overview of basic concepts in LC biochemical sensors, including physical properties of LC material, detection principles, and structure of the LC-based sensing platform that is classified into LC-solid, LC–aqueous, and LC droplet. Meanwhile, practical LC-based systems in detecting different targets are discussed on the basis of types of the analytes and reaction principle. The performance of different LC-based sensors is summarized and compared by using a table in each section. Furthermore, the combination of machine learning and LC-based sensors developed to enhance the performance of devices is presented as well. Finally, the challenges and future application prospects are concluded in the paper.

## 2. General Aspects of LC Sensors

### 2.1. Properties of LC

**Figure 1:**
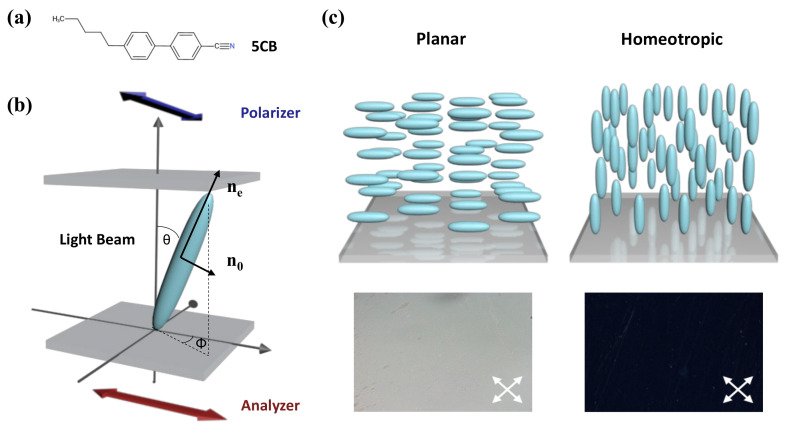
(**a**) The chemical structure of 5CB. (**b**) Schematic illustration of the orientational order of LC. (**c**) The planar and homeotropic alignment of LC at the surface and the corresponding images viewed under polarized optical microscopy (POM).

Among the different categories of thermotropic LCs, 4-cyano-4′-pentylbiphenyl (5CB) is the most commonly used component in LC-based sensing devices. This molecule contains a central biphenyl group and a cyano group, as depicted in Figure 1a. It has a stable nematic phase within 24 °C to 35 °C and enters an isotropic phase above the transition temperature T_N-I_ = 35 °C. The nematic phase processes a high degree of orientational order, but no positional order. As shown in the schematic diagram in Figure 1, the LC molecules prefer to align along the same direction spontaneously, which can be expressed by a unit vector, ***n***. The uniform direction leads to molecular anisotropy, the degree of which can be estimated by the order parameter, *S*:

(1) $$S=\frac{1}{2}\langle 3\cos^{2}\theta -1\rangle ,$$

where θ is the director angle against the long molecular axis of the LC molecule. The value of *S* equals 0 when LC is in an isotropic phase. When the LC is well aligned in the nematic phase, S approaches 1. Normally, its value fluctuates within a range of 0.3–0.8 upon the free energy variation [24].

In that case, the refractive index of LC is also anisotropic, resulting in optical anisotropy. It affects the velocity and phase of the light propagation through the medium. When the incident direction of polarized light is along the optical axis, LC exhibits an extraordinary refractive index $n_{e}$, while it shows an ordinary refractive index $n_{o}$ when perpendicular to the optical axis. The difference in the refractive index $\Delta n=n_{e}-n_{o}$ is defined as birefringence. When the polarized light beam passes at an angle θ against the direction of the LC director *n*, the effective refractive index will be changed into $n_{eff}$:

(2) $$n_{eff}=\frac{n_{e}n_{o}}{\sqrt{{\left(n_{e}\sin\theta \right)}^{2}+{\left(n_{o}\cos\theta \right)}^{2}}}$$

The differences in the LC system in orientation order and thickness will result in a difference in phase retardation of the incident polarized light, which can be expressed as δ:

(3) $$\delta =\frac{2\pi d}{\lambda }n_{eff}$$

where λ is the wavelength of incident light and *d* is the thickness of the LC film. The LC system is usually placed between two crossed linear polarizers: analyzer and polarizer. The intensity of transmitted light can be expressed by I:

(4) $$I=I_{0}\frac{1}{2}\sin^{2}2\phi \sin^{2}\frac{\pi d}{\lambda }n_{eff},$$

where ϕ is the azimuthal angle of the LCs against the surface and *I*_0_ is the light intensity before passing through the polarizers. In general, the orientation of LC molecules on the plane surface could be determined through the two angles θ and ϕ and classified into planar, tilted, and homeotropic alignment. It is worth noting that in the homeotropic state, θ=0° and I=0, resulting in a dark optical image. At the planar state, θ=90°, meaning bright images can be observed under crossed polarizers.

### 2.2. Detection Principle

Overall, the detection principle of the LC-based sensor is related to phase change or orientational change in LC, depending on the types of targets and geometries of the LC system. In this subsection, we will discuss two different sensing principles.

#### 2.2.1. Phase Change

The nematic-to-isotropic (N-I) phase transition behavior of LC can be triggered by volatile organic compounds (VOCs). In this regard, LC can be exploited as an optical transducer in the threshold sensing of VOCs [25]. The diffusion of vapors into bulk LC could disrupt the order of LC molecules, making it change from a nematic state to an isotropic phase. At the isotropic phase, LC exhibits fluidly without any birefringence. Therefore, this transition can be easily converted to a bright-to-dark optical change under POM. What is noteworthy is that this phase change is reversible, which means that isotropic-to-nematic phase transition will happen upon removal of the vapor source. However, this abrupt phase change may limit the continuous detection of VOCs; many strategies have been exploited to control the phase transition process. More discussion can be found in Section 4.4.

#### 2.2.2. Orientational Change

The orientational ordering of LCs is controlled by interfacial energetics, ranging from 10^−3^ to 1 mJ/m^2^, as shown in the literature [26,27,28]. The interfacial energetics of the LCs can be disturbed by events at the molecular level (nanoscale) on the surface and amplified into LC ordering change at the optical level (microscale) [29]. Most LC biochemical sensors are designed based on this principle, which then led to changes in the birefringence and optical appearance. Typically, the specific interaction between the recognition probe and analyte triggers the reorientation of LC. The tilting of LC molecules then leads to a change in the refractive index, so that it is amplified into colorful optical textures when observed under POM. The molecular interactions of the chemical or biological species vary from electrostatic interaction, covalent bonding, ligand coordination, specific binding events, enzymatic reactions, etc. Generally, these interfacial reactions occur at the LC–aqueous interface or solid substrate. Therefore, the LC-based sensing platforms contain three main categories: LC-solid platform, LC–aqueous platform, and LC droplet. In the next section, detailed introductions of the three platforms will be presented.

## 3. LC-Based Sensing Platforms

### 3.1. LC-Solid Platform

**Figure 2:**
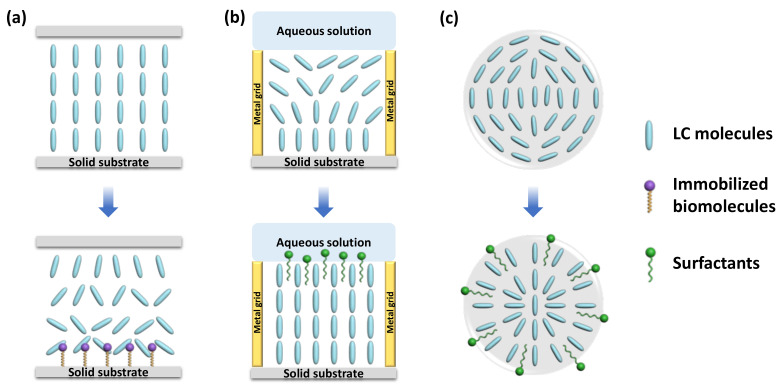
Schematic illustration of different types of LC-based sensing platforms. (**a**) LC-solid platform. (**b**) LC–aqueous platform. (**c**) LC droplet.

Surface anchoring effects rendered by solid substrates greatly affect the alignment of the LC supported on the substrates. This platform is typically made up of LC sandwiched between two glass substrates, named an LC cell. The fluid LC is often heated above the clearing temperature and injected into the LC cell by capillary force. After that, the LC is cooled down to room temperature from isotropic phase to nematic phase for sensing purposes. By introducing different surface-treatment methods, the LC can be planar, vertical, or tilted aligned [30]. Polyimide (PI) is often used as the planar alignment agent. The LC molecules can be aligned parallel on the mechanically rubbed PI layer. As for homeotropic alignment, some coupling agents, such as N, N-dimethyl-n-octadecyl-3-aminopropyltrimethoxysilyl chloride (DMOAP) and octadecyltrichlorosilane (OTS), are required to functionalize the substrate. The hydrocarbon chain of the binding agent molecules can provide anchoring energy and induce vertical orientation of LC molecules. Meanwhile, in some cases, the surface was also functionalized with recognition groups that can specifically react with the analytes through some chemical bonds, such as 3-aminopropyl-trimethoxysilane (APTES), glutaraldehyde (GA), and triethoxysilylbutyraldehyde (TEA). As illustrated in Figure 2a, upon some interactions between the probe and analyte, such as antibody–antigen binding, coordination interaction, and DNA hybridization, the initial homeotropic alignments of LC were disturbed. This rearrangement then led to the change in brightness and color in polarized optical images, indicating the presence and the concentration of the chemical or biological analytes.

### 3.2. LC–Aqueous Platform

LC is immiscible with aqueous solutions. Thus, a stable interface can be formed at the junction of the LC phase and aqueous phase. The alignment of the LC molecules at the interface relies on the type of molecules in the aqueous solution. For pure deionized water, LCs exhibit a planar orientation at the surface. However, with the existence of some surfactants (e.g., sodium dodecyl sulfate (SDS), cetyl trimethyl ammonium bromide (CTAB), hexadecyl trimethyl ammonium bromide (OTAB), dodecyltrimethylammonium bromide (DTAB)), biomolecules, and lipids (dilauroyl phosphatidylcholine (DLPC)) at certain concentration, the LCs have a tendency to be homeotropically aligned [15,31]. Since the surfactants and phospholipids are generally amphiphilic molecules, containing a hydrophilic group at one end and a hydrophobic group at the other end, the hydrophobic hydroxyl chain can penetrate through the LC phase and self-assemble at the interface, inducing the vertical alignment of LC molecules, as shown in Figure 2b.

Transmission electron microscopy (TEM) grid plays an important role in the LC–aqueous sensing platform. It is a grid made of copper or gold with hundreds of micron-scale meshes. The grid can be filled with LC to form a stable thin film [32,33]. In general, one side of the LC film in the TEM grid is supported on the glass substrate or contact with the air, where the LC molecules can achieve a vertical alignment; another side of the LC film is in contact with the aqueous solution. Without the presence of the surfactants or lipids, the LC film exhibits a hybrid orientation, giving a bright optical texture under the POM. The appearance of those specific molecules, however, promotes homeotropic orientation, giving a dark optical image under POM. This optical change can report the presence of the surfactants or lipids effectively.

Moreover, as some biomolecules show good affinity with surfactants, they can disrupt the balance between the surfactants and LCs and trigger the orientation transition from homotropic to planar. Based on this principle, more sensors can be designed for the detection of various targets, such as single-strand DNA, metal ions, and toxins, and so on [34,35,36]. Therefore, the LC–aqueous interface provides a functional, flexible, and real-time sensing platform for biochemical sensors.

### 3.3. LC Droplet

Another format of LC employed in LC-based sensors is droplets. LC droplets were dispersed in an aqueous solution, contacting with the surfactants or biomolecules in the aqueous phase. Many methods have been utilized to generate LC droplets, including emulsification, microfluidics, and inkjet printing. While emulsification is the simplest way to prepare LC emulsion droplets through shaking and ultrasonication, microfluidics is regarded as the best platform to generate uniform LC microdroplets with uniform size and desirable properties [37].

Because of large surface-area-to-volume ratio, diverse configurations, and special topological defects, LC droplets have become an ultrasensitive tool for LC sensing. Even tiny external changes at the surface of a droplet can induce the change in LC director and configuration. The configurations of the nematic LC droplets can be radial, pre-radial, or bipolar, as shown in Figure 2c. The formation of radial configuration is due to the perpendicular alignment of LC molecules to the surface of the droplet; the LC director points to the center of the droplet. For bipolar configuration, the interfacial LC molecules align parallel to the surface of the droplet and converge at the poles to form point defects. The pre-radial configuration is an intermediate conformation occurring during the transition from radial to bipolar configuration. When the target molecules adsorb onto the surface of the LC droplet and react with the recognition probe, it triggers the orientational change in LC molecules inside the droplets. This configuration transition can be then observed through POM directly or converted to changes in the whispering gallery mode (WGM) spectra. In the latter method, the LC droplets are regarded as optical resonators and possess WGM resonance, where the light is confined inside the surface by total internal reflection. The internal change in LC droplets in the refractive index can be reported by the shift in WGM spectrum [38].

## 4. Applications in LC Biochemical Sensors

Based on the versatility of LC sensing platforms, a great number of biological and chemical targets can be effectively detected. This section will focus on the practical applications of LC-based sensors in detecting specific target molecules, including their design mechanism, characteristics, detection performance, and signal transduction strategies.

### 4.1. Metal Ion Detection

Metal ions are essential to natural environments and biological systems. With the development of human society in recent decades, the release and accumulation of metal ions in water environments has become a serious issue, which is closely related to water quality and human health. Therefore, developing metal ion sensors is an important topic in bioanalytical chemistry. Although many methods have been used to detect metal ions with good accuracy, meeting industry standards (e.g., atomic absorption spectrometry, fluorescence spectrometry, electrochemical analysis), they generally require large and expensive equipment, as well as complex and time-consuming sample pretreatment processes. In contrast, LC-based metal ion sensors provide a sensitive, simple, fast, and low-cost method [22]. Table 1 summarizes the LC-based metal ion sensors developed in recent years.

**Figure 3:**
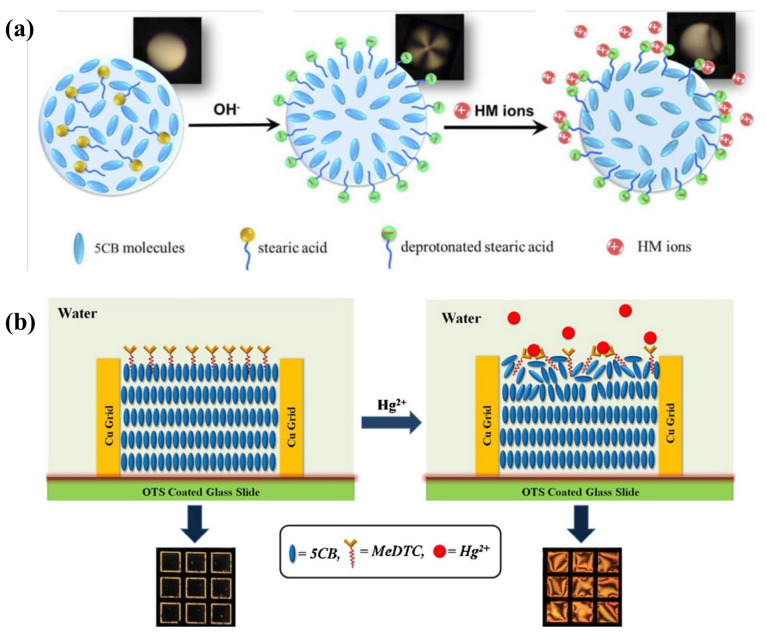
(**a**) Orientational transition of stearic-acid-doped 5CB microdroplets with the change in pH value and presence of heavy metal ions. Adapted with permission from Ref. [41]. 2019, Optical Society of America. (**b**) Hg^2+^ induced alignment changes in LC doped with MeDTC from a homeotropic to a planar state. Adapted with permission from Ref. [43]. 2016, Elsevier.

The detection of heavy metal ions in liquid crystals has been extensively studied, including Cu^2+^, Hg^2+^, Cd^2+^, Pb^2+^, etc. To detect Cu^2+^, the inhibition effect of urease activity by heavy metal ions was used as a model by Hu et al. Cu^2+^ inhibited the enzymatic hydrolysis of urea and kept the LC orientation at its original planar state from being vertically aligned by the release of ammonia [39]. Han et al. also reported a heavy-metal-ion sensor based on LC droplets doped with stearic acid, which was formed on the OTS-treated substrate with a homeotropic orientation. The addition of Cu^2+^ or Co^2+^ deprotonated the acid and led to a dark-to-bright transition [40]. To reduce the probability of misjudgment of naked-eye reorganization for the droplet pattern, Duan et al. demonstrated an alternative signal transducing method based on WGM lasing in a stearic-acid-doped LC droplet to detect heavy metal ions. With the increasing concentration of Cu^2+^, the WGM spectra shifted gradually with the change in the internal orientation of LC droplets (Figure 3a). They achieved quantitative detection of Cu^2+^ with LOD at 40 pM [41]. However, most of the detection probes for Cu^2+^ lack specificity as they exhibit affinity to other common heavy metal ions (e.g., Hg^2+^, Cd^2+^, and Zn^2+^) as well. The Cu^2+^-specific detection method should be further explored.

For the detection of Hg^2+^, Chen et al. doped the LC with a sulfur- and nitrogen-containing Hg^2+^ specific ligand 5-(pyridine-4-yl)2-(5-(pyridin-4-yl)thiophen-2-yl)thiazole (ZT), which formed a complex with Hg^2+^ in the aqueous solution, leading to an optical transition from dark to bright. It showed good selectivity and sensitivity at 10 μM [42]. Singh et al. developed a platform where the amphiphilic potassium N-methyl N-dodecyldithiocarbamate (MeDTC) self-assembled at the LC–aqueous interface with homeotropic alignment ability, as depicted in Figure 3b. It formed a chelate complex with Hg^2+^ to disturb the initial alignment and showed a bright texture. The limit of detection (LOD) of this Hg^2+^ sensing system was 0.5 μM [43]. However, the sensitivity of these sensing platforms using specific surfactants is still not satisfactory.

Apart from the application of metal ion specific chemical agents and formation of complexes, DNA molecules have been regarded as effective probes for metal ion detection due to their remarkable affinity and selectivity for metal ion binding. It provides ideal binding sites for metal via phosphate backbone and nucleobases. Moreover, DNA is stable, easy to synthesize, and highly programmable. The specific metal recognition DNA sequence is amenable to be selected in vitro, making it suitable for metal ion sensing. DNA-based sensors contain two types of working principles based on aptamers and DNAzymes.

**Figure 4:**
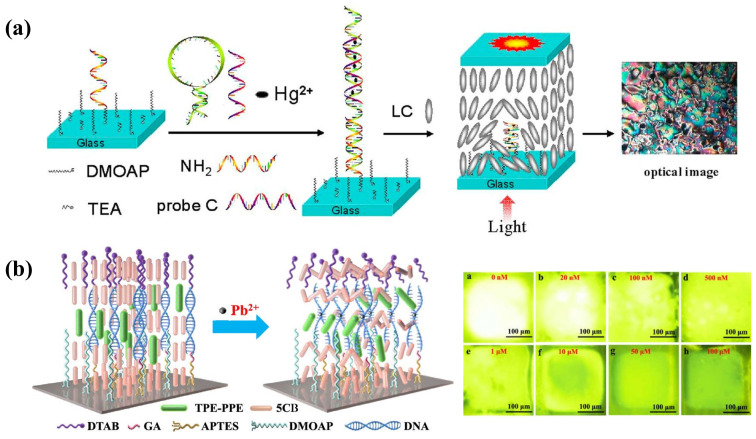
(**a**) Mechanism of the T–Hg^2+^–T-based LC sensing system for Hg^2+^ detection. Adapted with permission from Ref. [44]. 2012, American Chemical Society. (**b**) Schematic illustration of the fluorescence sensor for Pb^2+^ detection based on AIEgen-doped LC. The fluorescence intensity decreases with the increasing concentration of Pb^2+^. Adapted with permission from Ref. [48]. 2021, American Chemical Society.

As shown in Figure 4a, Yang et al. designed a novel Hg^2+^ sensing strategy with sensitivity up to 0.1 nM based on specific binding between Hg^2+^ and DNA thymine (T) base pairs. DNA duplexes are, thus, formed, containing T–Hg^2+^–T from the hybridization of a specific oligonucleotide probe and a complementary probe distorted the homeotropic orientation of LC into random orientation. The LOD of Hg^2+^ was reduced to 0.1 nM [44]. Furthermore, as Cd^2+^ has a good affinity with the guanine-rich DNA, Deng et al. proposed a Cd^2+^ sensor based on Cd^2+^-induced bending of the specific DNA (PS-Oligo) on the substrate, leading to the rearrangement of LC. It showed an obvious brightness change at a low concentrations of 0.1 nM [45]. Even though these methods achieved good sensitivity, the immobilization of oligonucleotides onto the substrate increases operational complexity.

Pb^2+^ can be detected with the assistance of a specific aptamer as well. Verma et al. selected a spinach RNA aptamer (SRNA) as a recognition element for Pb^2+^, which exhibits great affinity to form the G-quadruplex-shaped aptamer-Pb^2+^ complex. It was then utilized in the LC sensing platform. When the SRNA interacted with the cationic surfactant CTAB, the LC was planarly oriented. This interaction was disturbed with the presence of Pb^2+^ and the formation of an SRNA-Pb^2+^ complex. Then excess CTAB provided anchoring energy to LC to induce homeotropic alignment. By taking advantage of this strategy, they achieved a low detection limit of Pb^2+^ at 3 nM [35]. Duong et al. also reported an LC droplet Pb^2+^ sensor based on specific binding between DNAzyme and Pb^2+^. Initially, the cationic surfactant OTAB helps with the homeotropic orientation of LC. After the addition of DNAzyme, it transformed into a planar state. When Pb^2+^ was added into the system, a DNAzyme-Pb^2+^ complex formed and the conformation of DNAzyme changed. The orientation also returned to a homeotropic state due to the release of OTAB. The LOD of this LC droplet sensor was 0.7 nM [46]. Furthermore, Niu et al. developed an LC optical sensor based on the DNAzyme with a catalytic strand for Pb^2+^ detection. The complementary DNA strands were assembled at the APTES/DMOAP functionalized substrate. The homeotropic orientation of the LC film was obtained together with anchoring from DTAB in the water phase. In the presence of Pb^2+^, the catalytic DNA strand was cleaved and the complementary DNA unwound, leading to disordered configuration of LC [47]. However, the limit of detection at about 36.8 nM was not satisfactory. In order to improve its sensitivity, this group further imported the aggregation-induced emission luminogen (AIEgen) into the system; a Pb^2+^ fluorescence sensor based on AIEgen-doped LC was developed, as represented in Figure 4b. The mesogenic AIEgen can be aligned along the director of LC; the orientation change in LC was accompanied with a fluorescence change in AIEgen under UV irradiation. In the appearance of Pb^2+^, the catalytic hydrolysis of DNAzyme changed the alignment of LC and AIEgen, inducing the fluorescence signal change. This method resulted in better signal contrast and improvement in LOD at 0.65 nM compared to the traditional optical method [48].

The use of nanoparticles was also reported in Pb^2+^ sensing. Zehra et al. synthesized NiFe_2_O_4_ nanoparticles and applied them in an LC system. Due to the interaction between metal ions and hydroxyl groups at the surface of nanoparticles, a random orientation of LC and bright optical image were obtained. It showed higher selectivity to Pb^2+^, with LOD at about 100 ppb [49]. Nevertheless, the authors did not find a good linear relationship between Pb^2+^ concentration and the optical gray value to enable quantitative measurement. Thus, further investigation is required on the selectivity of the ferrite nanoparticles towards different heavy metal ions.

In addition to those heavy metal ions, the determination of the content of Ca^2+^, K^+^, and Na^+^ in human serum is also essential. It can provide a basis for replenishing electrolyte, maintaining osmotic pressure, and acid–base balance of body fluids. Cholesteric liquid crystal (CLC) has been reported as an optical sensor for metal ion sensing due to its unique photonic structure. The sensing platform was formed by incorporating some special coordination moieties that respond specifically to different metal ions. Kado et al. made use of the crown ether derivative as the K^+^ recognition site to incorporate with a CLC film to induce the reflection-peak shift [50]. In the continuous work, Stroganov et al. modified the membrane into polymer-stabilized CLC to stabilize the crown-ether-containing LC film and prevent it from dewetting. It showed a blue shift not only in the presence of K^+^, but also Ba^2+^ [51]. Taking the stronger binding affinity of Ca^2+^ to carboxylates compared with other bivalent ions into consideration, the system containing a carboxylic acid group (-COOH) was demonstrated to be applied in Ca^2+^ detection [52]. The Ca^2+^ treatment can lead to the formation of a bridged structure between carboxylic groups (R_1_–COO–Ca–OOC–R_2_), disrupting the original structure of the sensing system. Moirangthem et al. introduced benzoic-acid-functionalized molecules to fabricate the chiral imprinted CLC polymer film. After treating with KOH, it was converted into polymer potassium film to be used for Ca^2+^ sensing. The exchange reaction of K^+^ by Ca^2+^ led to a decrease in helical pitch length and an apparent blue shift in the range of 10^−4^ and 10^−2^ M [53]. Myung et al. also reported an interpenetrating polymeric network consisting of a cross-linked CLC network and a poly(acrylic acid) (PAA) network to determine the amount of Ca^2+^. This acetone/KOH-treated photonic film exhibited a rapid optical response to humidity and Ca^2+^. The naked-eye measurement of Ca^2+^ from 0.35 to 3.4 mM was able to be achieved [54]. Overall, the CLC photonic film-based sensing system shows great potential in monitoring the amount of metal ions in human serum.

On top of that, another Ca^2+^ detection method using LC combined with a block copolymer poly(acrylicacid-b-4-cynobiphenyl-4-oxyundecylacrylate) (PAA-b-LCP) was successfully achieved. The LCP block linked with the Ca^2+^-responsive PAA chain was anchored to 5CB supported on the glass substrate. The appearance of Ca^2+^ in the NaCl solution induced the homeotropic-to-planar orientational change due to the replacement of Na^+^ with Ca^2+^ in the PAA chain [55]. However, the LOD was relatively high at 2.5 mM. Since it could also respond to many other divalent ions, such as Cu^2+^ and Mg^2+^, the selectivity is not satisfied. Therefore, more efforts should focus on strategies to improve the LOD and selectivity of Ca^2+^ sensors. What’s more, the LC-based sensor for Na^+^ detection was seldom reported. Continuous studies need to explore the recognition probe for Na^+^.

**Table 1:** LC sensors for metal ion detection.

LC Material	Sensing Platform	Analytes	Detection Probe	Principle	LOD	Ref.
UV-treated 5CB	LC–aqueous	Cu^2+^	Urease	LC keep the orientation when urea contacts with the Cu^2+^-blocked urease	10 μM	[39]
Stearic-acid-doped 5CB	LC droplet	Cu^2+^/Co^2+^	Stearic acid	HM ions attached to the deprotonated acid, interrupt the self-assembly of stearic acid and LC orientation	0.3–0.5 nM	[40]
Stearic-acid-doped 5CB	LC droplet	Cu^2+^	Stearic acid	Spectral shift of WGM with the adsorption of HM ions on 5CB microdroplet	40 pM	[41]
Fluorophore/5CB	LC-solid	Cu^2+^	Biotinylated oligopeptide	Combination between multidentate oligopeptide ligand Cu^2+^, inducing LC reorientation	0.1 μM	[56]
ZT-doped 5CB	LC–aqueous	Hg^2+^	ZT	Complexation between ZT and Hg^2+^, inducing LC reorientation	10 μM	[42]
5CB	LC–aqueous	Hg^2+^	MeDTC	Complexation between MeDTC and Hg^2+^, inducing LC reorientation	0.5 μM	[43]
5CB	LC-solid	Hg^2+^	T-T base pairs	Formation of DNA duplexes containing T–Hg^2+^–T, inducing LC reorientation	0.1 nM	[44]
5CB	LC-solid	Cd^2+^	PS-Oligo	Cd^2+^ induced bending of PS-oligo, inducing LC reorientation	0.1 nM	[45]
5CB	LC–aqueous	Pb^2+^	SRNA	Formation of SRNA-Pb^2+^ complex, inducing LC reorientation	3 nM	[35]
5CB	LC droplet	Pb^2+^	DNAzyme	Binding of Pb^2+^ and DNAzyme releases OTAB, inducing LC droplets order transition	0.7 nM	[46]
5CB	LC-solid/aqueous	Pb^2+^	DNAzyme	Catalytic hydrolysis of DNAzyme by Pb^2+^, inducing LC reorientation	36.8 nM	[47]
AIEgen-doped 5CB	LC-solid/aqueous	Pb^2+^	DNAzyme	Catalytic hydrolysis of DNAzyme by Pb^2+^, inducing LC/AIEgen reorientation and fluorescence change	0.65 nM	[48]
5CB	LC–aqueous	Pb^2+^	NiFe_2_O_4_ nanoparticle	Interaction between Pb^2+^ and -OH on the nanoparticle, inducing LC reorientation	100 ppb	[49]
CLC	CLC film	K^+^	Crown ether derivative	Binding between K^+^ and crown ether derivative, inducing reflection peak shift	120 nm M^−1^	[50]
CLC	PSCLC film	K^+^/Ba^2+^	Crown ether	Binding between K^+^/Ba^2+^ and crown ether, inducing reflection peak shift	\	[51]
CLC	CLC film	Ca^2+^	Benzoic acid group	Coordination interaction between Ca^2+^ and -COO^−^, inducing reflection peak shift	10^−4^ M	[53]
CLC	CLC film	Ca^2+^	Poly(acrylic acid)	Coordination interaction between Ca^2+^ and -COO^−^, inducing reflection peak shift	0.35 mM	[54]
5CB	LC–aqueous	Ca^2+^	PAA-b-LCP	Coordination interaction between Ca^2+^ and -COO^−^, inducing LC reorientation	2.5 mM	[55]

### 4.2. Nucleic Acid Detection

**Figure 5:**
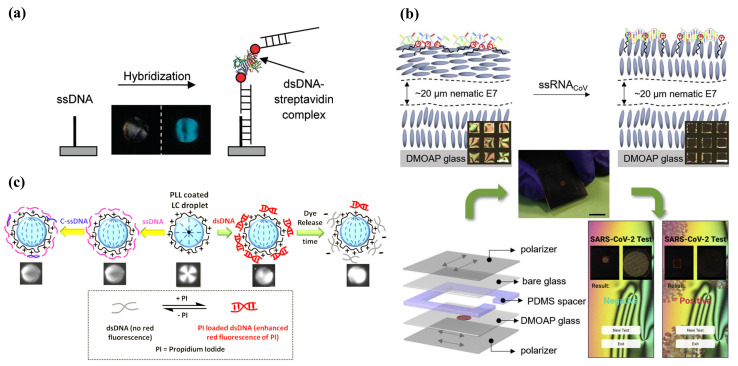
(**a**) The change in optical image by DNA hybridization. Adapted with permission from Ref. [64]. 2011, American Chemical Society. (**b**) General sensing mechanism and set-up of an LC-based diagnostic kit for SARS-CoV-2 detection. Test result readout by smartphone app for negative and positive test results. Adapted with permission from Ref. [72]. 2020, Elsevier. (**c**) PLL-coated LC droplets in radial configuration and change into bipolar configuration change with the absorption of ssDNA or dsDNA. Adapted with permission from Ref. [75]. 2017, American Chemical Society.

Nucleic acids can be classified into DNA and RNA. They are biological molecules in the cell that carry the genetic information of the living organisms, which are essential in protein synthesis and keeping the normal function of living bodies. Therefore, the detection of nucleic acid plays an important role in the biological and medical fields, including gene analysis, rapid screening of virus, early diagnosis of diseases, forensic science, and so on [57]. A great number of DNA biosensors based on hybridization between DNA or RNA targets and complementary probes have been developed through optical or electrochemical methods [58,59,60]. Otherwise, DNA microarrays have also been regarded as an alternative tool for continuous, fast, and sensitive detection of DNA hybridization. In DNA microarrays, DNA probes are always immobilized onto the glass or silicon supports. Therefore, this scheme can be applied in LC sensing platforms as well. The characteristics of DNA strands greatly affect LC orientation, including molecular length, average density and surface coverage, and hybridization state. Kim et al. demonstrated the oligoDNA immobilized a biotin chip to distinguish single- and double-strand DNA through the anchoring transition of LC. After the hybridization, LC transformed from a homeotropic to a random alignment, leading to bright images when observed by the naked eye [61]. Lai et al. also studied the behavior of DNA with different bases absorbed on solid substrates and built DNA microarrays to achieve quantitative detection of DNA through LC imaging [62,63]. They further utilized the DNA–protein complexes to enhance the optical signal in the system; the DNA hybridization-induced LC signal change was observed more clearly (Figure 5a) [64]. Moreover, as the negatively charged DNA can be a complex with metal ions, this principle was used to establish an LC sensor for DNA target detection by Liu et al. When DNA contacted with Na^+^, the complex disturbed the orientation of LC. In contrast, the electroneutral peptide nucleic acid (PNA) still produced a dark image with the presence of Na^+^, making it become a probe to capture a specific DNA target. It achieved 10 fM LOD of the 600 bp DNA target [65]. Shen et al. proposed a new TEA-modified substrate to immobilize ssDNA probes, with which the LC formed a homeotropic alignment at certain concentration. Once the complementary DNA hybridized with the probe, the LC became randomly aligned. This system was reported to have an LOD of about 0.1 nM for DNA analytes [66]. The DNA microarray technology provided a label-free, large-scale, and high-throughput method for nucleic acid detection. However, the sensitivity of the bioassay technique in LC sensing is limited by the amount and size of the biomolecules. It is necessary to explore new strategies for signal enhancements. Tan and co-workers demonstrated a signal-enhanced LC DNA biosensor based on enzymatic metal deposition for the first time. The overall immobilized DNA system was formed by a capture DNA probe, a target DNA, and a biotinylated detection DNA probe. The biotin bound to streptavidin alkaline phosphatase (Sv-ALP), which served as a catalyst to hydrolyze the ascorbic acid 2-phosphate (AA-p) into ascorbic acid. Then, Ag^+^ was reduced by ascorbic acid into metallic silver and deposited on the substrate. Hence, the sensitivity of the LC sensor was closely related to the enzymatic reaction and a decrease in LOD from 1 nM to 0.1 pM was contributed by silver deposition [67]. This enzymatic signal enhancement approach provided a new route for the detection of other biomolecules.

In addition, the LC–aqueous interface was also set up as a DNA sensing platform. It is well known that the anionic DNA can interact with cationic surfactants in aqueous solutions to form insoluble complexes. However, the single-strand DNA (ssDNA) and double-strand DNA (dsDNA) have different contributions to the LC–aqueous interface due to electrostatic and hydrophobic effects [68]. Based on this principle, McUmber et al. investigated the change in LC orientation at the surfactant-ladened interface after the absorption of ssDNA or dsDNA, respectively. When the ssDNA absorbed at the surfactant-ladened interface, it led to LC reorientation from homeotropic to planar. Subsequently, the exposure of the ssDNA–surfactant complex to the complementary ssDNA led to the formation of dsDNA and the release of surfactants. Therefore, the DNA hybridization allowed the LC to go back to the homeotropic state, which can be reflected by the bright-to-dark change in the LC optical image [69,70]. More importantly, this LC-based DNA sensor played an important role in gene or pathogen detections. Tan et al. reported a specific sensing strategy for a p53 mutation gene segment through the absorption of DNA dendrimer at the LC–aqueous surface, which induced the tilted-to-homeotropic orientational change in SDS-doped LC. The concentration of the target required to obtain the signal change ranged from 0.08 to 8 nM [71]. Khan et al. designed an LC-filled TEM grid for detecting genomic DNAs of bacteria and fungi. The ssDNA probe was absorbed onto the DTAB-functionalized LC–aqueous interface. A planar-to-homeotropic orientational change could be triggered after it was exposed to ssDNA targets. It has a sensitivity of 0.05 nM and good selectivity to the specific ssDNA targets compared to mismatched DNA [34]. Similarly, this ultrasensitive and selective method was applied to SARS-CoV-2 RNA detection by Xu et al. as shown in Figure 5b. They found that the ssRNA of SARS-CoV-2 could pair with a complementary ssDNA probe and generate order change in LC. The dark image under POM appeared when the concentration of the ssRNA target was about 30 fM [72].

Moreover, the assembly behavior of DNA amphiphiles at the LC–aqueous interface was exploited for DNA detection. Lai et al. designed a cholesterol-labeled DNA probe that can induce a homeotropic alignment of LC. When it was hybridized with a complementary DNA target, the LC orientation was tilted [73]. On the contrary, it was found by Zhou et al. that the DNA–lipids could not trigger LC homeotropic alignment. Based on this property, they co-assembled DNA–lipids and L-DLPC at the interface, where L-DLPC was located at the entire surface and DNA–lipids located at the net-like structures. After the DNA–lipids hybridized with complementary DNA, it disassembled from the surface and only L-DLPC remained. This transformation was indicated by the change in optical images from net-like structures to dark with bright ellipsoidal domains [74]. As for the detection of DNA by LC droplets, it was first investigated by Verma et al. They fabricated the poly(L-lysine) (PLL)-coated LC droplets, which can interact with ssDNA or dsDNA and trigger the change in configuration of LC droplets from radial to bipolar, as depicted in Figure 5c. This LC-droplet-based detection system was also found to be employed as a nanocarrier for controlled drug release [75]. The works related to LC sensors for nucleic acid are listed in Table 2.

### 4.3. Protein Detection

Protein is one of the most important substances in living organisms, which is the basic building block of cells and tissues. There are various kinds of proteins in living organisms, with different properties and functions. Therefore, the specific detection of proteins is extremely important for health monitoring, clinical testing, disease diagnosis, and prognosis. LC-based biosensors for protein detection have been studied for years and are now a mature system, which is summarized in Table 3. Taking advantage of the good biocompatibility of LC, the interaction between proteins, or specific ligand–protein can be amplified through LC.

#### 4.3.1. Immunoreaction-Based Sensing

In general, proteins with good antigenicity can be determined by antigen–antibody reactions. This specific reaction will generate an immunocomplex at the aqueous interface or on the modified substrate, then lead to the reorientation of LC molecules, giving rise to a bright image under POM.

**Figure 6:**
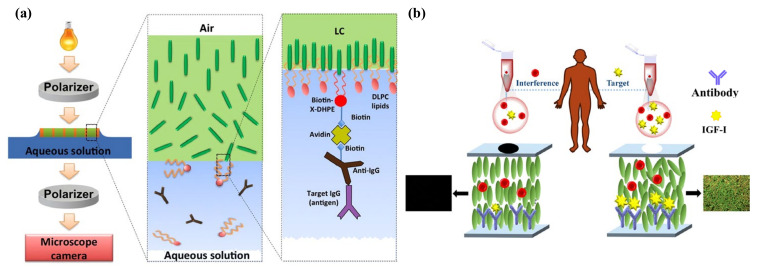
(**a**) Schematic illustration of LC–aqueous sensing platform for IgG detection. Adapted with permission from Ref. [76]. 2016, Elsevier. (**b**) Schematic illustration of IGF-I diagnosis by LC-based immunosensor. The specific binding between IGF-I and its antibody leads to LC reorientation. Adapted with permission from Ref. [91]. 2021, Elsevier.

For instance, the goat Immunoglobulin G (IgG) was sensed through a suspended LC film coated with biotinylated anti-goat IgG by Popov et al. The experiment set-up and scheme are shown in Figure 6a. The biotinylated lipids formed a link between target IgG and anti-IgG. Only when the goat IgG appeared in the solution, the specific immunoreaction could result in a rapid dark-to-bright optical change [76]. Zhu et al. developed a microfluidic immunoassay with LC flow inside for rabbit IgG detection. The anti-IgG was firstly immobilized at the channel wall, on which LC oriented homeotropically. When the antigen IgG bound with the antibody specifically, a dark-to-bright optical change appeared due to the disruption in LC orientation [77]. Lee et al. conducted research on the detection of IgG by using LC microdroplets. SDS and a poly(styrene-b-acrylic acid) copolymer (PS-b-PA) were used to modify the LC–aqueous interface and stabilize LC molecules in a radial configuration. The anti-IgG was anchored on the LC droplets with the activated carboxylic group. After interacting with the IgG antigen, the radial-to-bipolar transition was observed. The formation of an anti-IgG-anchored LC microdroplet allowed the detection of the IgG antigen at a detection limit of 16 ng/mL in 30 min in PBS solution [78,79]. Furthermore, Huan et al. proposed a glass immobilization method to capture anti-IgG-conjugated LC microdroplets to detect IgG antigens. This strategy was designed to enhance the interactions between anti-IgG and IgG, as well as the stability in antigen detection in the biological fluids [78,79].

Carbohydrate antigen 125 (CA125) is a glycoprotein that is commonly used as a tumor marker for ovarian epithelial carcinoma. A series studies was carried out on the label-free CA125 immunodetection based on LC by Su et al. They immobilized anti-CA125 antibodies on the UV-modified DMOAP substrate to enhance its binding affinity with CA125 [80]. The bright optical texture obtained under crossed polarizers was correlated with the CA125 concentration. Furthermore, they also explored different LC materials to improve the sensitivity to about 0.1 ng/mL, such as high-birefringence LC and lyotropic chromonic LCs [81,82]. Similar immunoassays were conducted for the detection of many other proteins, including ischemia-modified albumin (IMA), bovine serum albumin (BSA), cardiac troponin I (cTnI), and so on [83,84,85,86]. He et al. proposed an IMA sensing method based on LC, which is for clinical diagnosis of myocardial ischemia. DMOAP and polyetherimide (PEI) served as a self-assembled layer to anchor the anti-IMA antibody. The binding between IMA and anti-IMA led to changes in surface topography, accompanied with the LC orientational change. The lowest concentration of IMA that could be detected was 50 μg/mL [83]. Fan et al. fabricated a polydimethylsiloxane (PDMS)-based multi-microfluidic LC immunoassay to report the behavior between BSA antigen–antibody pairs. It showed a naked-eye detection of the immunocomplex through optical signal, with an LOD down to 0.01 μg/mL [84]. Meanwhile, Huang and co-authors developed a new dye-doped LC (DDLC)-bioinspired sensor for colorimetric immunodetection of BSA. DDLC was made up of LC E7 and a red dichroic dye, which has absorption anisotropy (dichroism) in LC. The disruption of LC alignment by the BSA antigen–antibody pair was described by the color intensity. By employing the software on a smartphone to capture the colored images, the LOD at 0.5 ng/mL was achieved [85]. It is meaningful to expand the study on the composite of non-LC (e.g., nanomaterials, dichroic dye, chiral dopants, polymer) and LC materials to improve the performance of LC immunosensors [87,88].

The binding efficiency and affinity of the proteins on the substrate are closely related to the signal intensity and performance of the LC-based biosensor. In terms of surface decoration, the APTES/DMOAP mixed layer functionalized with GA has been widely exploited to immobilize antibodies. While DMOAP with long alkane thiols mainly helps with the vertical alignment of LC, APTES has short alkane thiol with amino groups to react with aldehyde groups of GA, which acts as the crosslinking agent to immobilize the antibodies. Zhang and Su et al. reported the detection of cecropin B (CB) and human β-defensin-2 (HBD-2) through this mixed self-assembled monolayer successively. The LC cell was fabricated by covering this functionalized substrate with a DMOAP-coated slide and filled it with 5CB. Upon the specific binding between the anti-CB antibody and CB, or anti-HBD-2 antibody and HBD-2, the orientation of LC changed from homeotropic to tilted, increasing the brightness of the polarized optical image. This label-free detection method provided a low LOD for CB at 50 ng/mL and HBD-2 at 0.53 ng/mL, respectively [89,90]. In addition, Xia et al. proposed an LC-based cardiac troponin I (cTnI) immunosensor. cTnI plays an important role in the diagnosis of acute myocardial infarction. By using a double-side functionalized substrate with DMOAP/APTES/GA and anti-cTnI antibody, they achieved 1 pg/mL detection limit of cTnI antigen [86]. Very recently, Chen and co-workers verified the use of LC in sensing human insulin-like growth factor-I (IGF-I), which is a polypeptide hormone that is responsible for the diagnosis of growth hormone (GH)-related diseases. As shown in Figure 6b, IGF-I antibody (Ab_IGF-I_) performed as the recognition probe being immobilized on the DMOAP/APTES/GA modified substrate. The complexation of IGF-I antigen (Ag_IGF-I_) and Ab_IGF-I_ gave rise to a gradual alignment change in LC and light intensity change. It showed a sensitive detection of Ag_IGF-I_ with LOD of 1.0 ng/mL and linear range of 10–2000 ng/mL [91].

For some applications, it is necessary to detect many disease-related proteins or peptides. They function as biomarkers for corresponding diseases. For example, Kim et al. fabricated a nanostructured substrate to immobilize anti-tuberculosis (TB) to diagnose TB in clinical specimens through LC. When it was incubated with the clinical serum samples from TB patients, a random bright image was observed. Yet, the clinical serum samples from healthy people did not induce optical changes [92]. Kemiklioglu et al. achieved amyloid-beta-42 (Ab42) detection, the level of which is associated with Alzheimer’s disease (AD). The immunoreaction between the Ab42 antibody and peptide occurred on a DMOAP-coated surface. Afterward, Apolipoprotein E4 (ApoE4), another risk factor for AD, was added into the system to form a triadic complex, changing the optical textures and reflectance of the LC film. The lowest concentration of Ab42 that could be detected was 1 pg/mL and the detection range of ApoE4 was 0.1–30 nM through this system [93].

To date, nanoparticles have usually been adopted in LC sensing platforms to enhance the signal and improve sensitivity. Nanoparticles made of gold, nickel, and silver have been used in LC sensing platforms [67,94,95,96]. As for immunoreaction-based sensing, gold nanoparticles (AuNPs) were integrated with an immunocomplex to be fixed onto the substrate. Due to the larger size of AuNP, it can induce stronger disruption on the LC molecules and give signal amplification. He’s group and Chen’s group reported the AuNP-assisted detection of cecropin B (CB) and protein kinase C (PKC), respectively. Their detection mechanism was similar, based on immunocomplexes and AuNPs’ induced LC orientational change. The LODs were improved to 1.49 × 10^−2^ ng/mL for CB and 1.68 ng/mL for PKC [96,97], respectively. The LC-based immunosensor shows great potential in point-of-care testing for clinical diagnosis. It can be further developed into a simple, rapid, and portable detection instrument.

#### 4.3.2. Enzymatic-Reaction-Based Sensing

An enzyme is a kind of protein produced by living cells, with high specificity and high catalytic efficiency to a biological substrate. As an important biocatalyst, the activity of enzymes is strongly associated with the biological metabolism, nutrition conversion, and energy transfer processes in living organisms. Meanwhile, it plays an increasingly significant role in disease diagnosis, clinical treatment, and industrial production. Hence, it is meaningful to detect enzymes and their activities. Among the various methods to detect enzymes and enzymatic reactions, LC-based biosensors are regarded as a promising candidate.

Urease, an enzyme that catalyzes the hydrolysis of urea to ammonia and carbon dioxide, has many applications in both medical and agriculture fields. It can be used to assess the bacteria level in the human body or environmental conditions [98]. Hu et al. reported the real-time screening of urease through 4-cyano-4′-biphenylcarboxylic acid (CBA), generated by UV-treated 5CB. The deprotonation of CBA was triggered by the release of ammonia from a urease-catalyzed reaction. With the ionization of ammonia into hydroxide (OH^−^) and ammonium ions (NH_4_^+^) in an aqueous solution, the deprotonation of CBA was facilitated and the repulsive interaction between the carboxylate head groups was shielded. Then, the area density of CBA at the LC–aqueous interface increased, inducing the orientational transition of LC from planar to homeotropic. The lowest urease concentration to be detected was 1 nM in this system [99]. On the basis of this principle, Liu et al. developed a simpler method for imaging urease activity with LC droplets on solid substrates. The stearic-acid-doped LC droplets exhibited planar orientation in urea or urease solution. Once urea and urease were mixed, the LC droplets had a perpendicular orientation and dark patterns were observed [100]. Qi et al. introduced stimulus-responsive nanocomposites to develop a new LC sensor for monitoring urease activity. The pH-responsive surfactant-encapsulated phosphotungstate clusters (SECs) were deposited on the substrate, on which the LC layer was assembled. Upon addition of the solution containing urea and urease to the LC, a bright appearance was displayed due to the disassembly of SECs with the increase in pH, which was induced by urea hydrolysis. Thus, the urease activity was sensed as low as 0.03 mU/mL [101]. Remarkably, the urease detection system is also able to determine the amount of heavy metal ions (e.g., Cu^2+^), which often serve as the inhibitor in the enzymatic reaction to block the active site of urease [99,102]. When Cu^2+^ was added into the urea and urease mixture, the LC image returned to a dark appearance due to the inhibition of urease activity [101].

**Figure 7:**
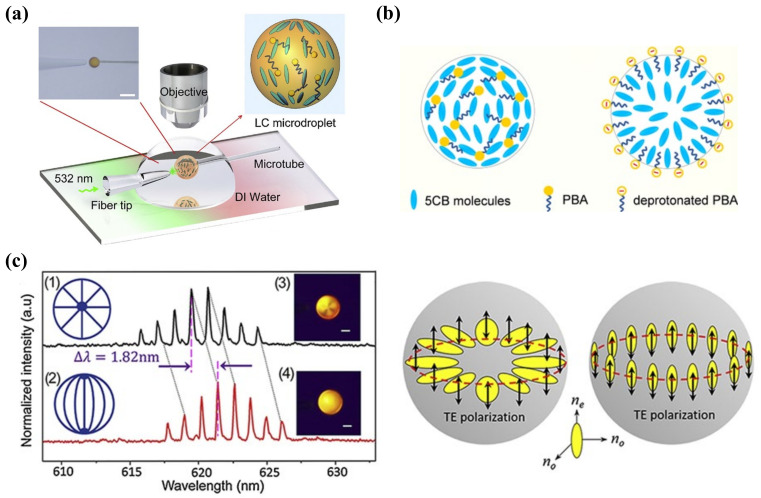
(**a**) Experiment setup of observing the WGM lasing emission in microdroplets under a pulse laser pump. (**b**) Structural transition of PBA-doped 5CB microdroplets at pH = 5.7 and 6.0. Adapted with permission from Ref. [106]. 2018, Elsevier. (**c**) WGM lasing of 5CB microdroplets with (1) (3) radial and (2) (4) bipolar configurations and a schematic illustration of the TE polarization where the electric field oscillates parallel or perpendicular to the microdroplet surface. Adapted with permission from Ref. [105]. 2020, Elsevier.

Many LC-based enzyme sensors are in a format where surfactants are doped with LC or decorated at the LC–aqueous interface, which are sensitive to the enzymatic hydrolysis process or the products. For example, Hu et al. selected dodecanal-doped LCs for catalase detection based on the oxidation of aldehyde by hydrogen peroxide into carboxylic acid. The formation of a carboxylate monolayer at the interface resulted in a homeotropic state for LC. However, the presence of catalase accelerated the hydrolysis of hydrogen peroxide, making the LC remain at planar state [103]. Owing to the great significance to the central nervous system of detecting AchE and its inhibitors, several LC-based sensing methods were carried out. Wang et al. constructed a cationic surfactant, myristoylcholine chloride (Myr)-decorated LC platform for acetylcholinesterase (AchE) detection. The hydrolysis of Myr by AchE was coupled with LC orientational transition, followed by dark-to-bright optical transition. It could not only detect AchE at 0.000827 U/mL, but also detect its inhibitor with high sensitivity at 1 fM [104]. By using the same reorganization probe Myr, Duan et al. constructed an LC droplet sensor for detecting AchE and its inhibitors. Upon the catalytic cleavage of Myr by AchE, the molecular orientation of the LC droplet gradually transferred from radial to bipolar. This change was further enhanced by the WGM spectral resonance, giving it great potential in sensing harmful pesticides [105]. The WGM of LC microdroplets was applied in detecting enzymatic reactions in penicillinase as well. Wang’s group found that 4′-pentyl-biphenyl-4-carboxylic acid (PBA)-doped 5CB showed a bipolar-to-radial response with the increase in pH from 5.7 to 6.0 due to the deprotonation of PBA. In that case, the decrease in pH value as well as the production of proton (H^+^) from penicillin G hydrolysis resulted in the radial-to-bipolar configuration transition and the redshift in WGM lasing spectra [106]. Therefore, it can be seen in Figure 7 that the pH-responsive LC microdroplet-accompanied WGM spectrum can take responsibility in monitoring many enzymatic-reaction-associated pH changes. Despite the WGM lasing spectrum being an ultra-sensitive method to report the internal orientation in LC droplets, some challenges in operation, signal read-out, and the thermal effect of the lasing on the LC droplets remained.

Wang et al. also successfully established a surfactant-LC system for detecting cellulase and cysteine (Cys). The dark-to-bright transition was attributed to the dodecyl β-d-glucopyranoside hydrolyzed by cellulase. By taking account of the chelating effect between Cu^2+^ and Cys that can prevent cellulase from being inhibited by Cu^2+^, this system was capable of recognizing the presence of Cys if the LC image maintained its brightness. The LOD of cellulase and Cys was about 1 × 10^−5^ mg/mL and 82.5 μM, respectively [107]. In the meantime, Zhou et al. doped a cleavable surfactant, N-octadecyloxycarbonylmethyl-N,N,N-trimethylammonium bromide (OTB) with 5CB to prepare a carboxylesterase (CES) sensor. OTB formed a monolayer at the LC–aqueous interface to induce a vertical alignment of LC. The addition of CES hydrolyzed OTB, leading to disruption in the monolayer and planar orientation of LC. The LOD of the sensor was reported as 18 U/L [108].

Likewise, the lipid-decorated LC layer was usually served as a sensing element for enzymatic activities. Hussain et al. presented a phospholipid-functionalized LC monolayer to sense the lipase activity, thereby measuring cell viability. The disorder of LC was triggered by the phospholipase catalysis of a phospholipid and a bright image was observed [109]. To avoid the use of POM, Tang et al. incorporated LC into the optical fiber to detect phospholipase A_2_ (PLA_2_), which was fabricated by coating 5CB and phospholipid (L-DLPC) outside the side-polished fiber (SPF). When the functionalized SPF was immersed in the solution containing PLA_2_, the hydrolysis between PLA_2_ and L-DLPC redistributed the order of 5CB and altered the *n_eff_*, inducing a change in the transmission optical power. This novel fiber sensor has a very low LOD at 1 nM [110]. This optical fiber sensor incorporated with LC can also be further studied to detect more biomolecules and developed into POM-free biosensors.

Trypsin is a digestive enzyme that maintains the function of the digestive system. Several studies have been carried out to monitor the trypsin activity. Hu et al. made use of the polyelectrolyte, PLL, and the phospholipid, dioleoyl-sn-glycero-3-phospho-rac-(1-glycerol) sodium salt (DOPG), assembled LC as the initial planar state. In the presence of trypsin, the enzymatic reaction of trypsin and PLL drove the planar-to-homeotropic orientational transition. Its LOD was determined at about 1 μg/mL [111]. By replacing PLL with poly-L-arginine hydrochloride (PLA), which specifically reacted with a serine protease thrombin, Zhang et al. also achieved the detection of thrombin through LC [112]. Moreover, Chuang et al. reported the BSA-modified grid filled with LC for trypsin detection. The BSA was cleaved by trypsin and released peptide fragments, which disrupted the orientation of LC and generated a bright optical image. This method obtained a lower LOD at 10 ng/mL compared with the previous monolayer-based method [113]. Specially, a novel integrated LC-based trypsin sensing device was designed by Ping et al. by mixing gelatin hydrogel with surfactant CTAB. The gelatin decomposed with the addition of trypsin, thereby releasing the embedded CTAB to induce homeotropic alignment of LC. It had a remarkable LOD at 0.34 ng/mL, showing great potential in the clinical detection of trypsin in human serum [114].

In order to study the protease activity, Jannat and co-authors proposed a continuous LC-based assay where proteases cleaved casein into small peptide fragments and absorbed on the surface. It then disrupted the LC orientation, producing bright spots for naked-eye detection. Through this assay, 10 ng/mL of protease was detected and protease kinetic study was performed [115]. Subsequently, they built a protease inhibition assay in a millifluidic device to learn the inhibition effect of pefabloc irreversibly. The half maximal inhibitory concentration (IC_50_) of pefabloc for the protease was found to be 0.45 mg/mL [116]. This LC-based continuous assay was useful for analyzing the enzyme activity as well as the inhibiting effect.

#### 4.3.3. Aptamer-Based Sensing

The aforementioned discussion suggests that aptamers can be customized and specifically bind to targets, which has functional equivalence with antibodies. However, it is hard to obtain the antibodies for the molecules that are too small or have poor immunogenicity or high toxicity. Aptamers show certain superiority over protein-based probes in sensing fields. They can be selected and prepared in vitro, undergo chemical modifications, and specifically bind to low-molecular-weight molecules [117]. Therefore, there are a great number of aptamers being employed to bind with analytes, including enzymes, peptides, or viral proteins. Kim et al. proposed the LC-based aptasensor for thrombin detection. The thrombin-specific aptamers were immobilized on the substrate through APTES/GA binding, inducing homeotropic alignment of LC with the assistance of DMOAP. Interaction between thrombin and its aptamer made LC become disordered. This aptasensor has high specificity as well as high sensitivity at 1 pg/mL [118]. Meanwhile, Ren and Kim developed the LC-aptamer detection system for carcinoembryonic antigen (CEA), a tumor marker, and interferon-γ (IFN-γ), a cytokine critical to immunity, respectively. Both the CEA aptamer and IFN-γ aptamer were immobilized onto the APTES/DMOAP-coated surface as molecule identification elements. The binding between target protein and its aptamer gave a dark-to-bright image transition. The minimum concentrations to be detected were 0.12 pg/mL for CEA and 17 pg/mL for IFN-γ, respectively [119,120].

Alpha-synuclein (α-syn) is a protein that plays an important role in the pathogenesis of Parkinson’s disease (PD). A DNA aptamer was found with specific binding ability with α-syn; thus, an LC-based aptasensor was fabricated to test the level of α-syn by Yang et al. The aptamers were anchored through thiol–gold linkage on gold-coated glass, which was modified with a self-assembled monolayer (SAM) to induce initial homeotropic alignment of LC. When the α-syn appeared, it were captured by its aptamer, inducing the realignment of LC. The LOD of this α-syn sensor was 50 nM [121]. Abbasi et al. utilized an RNA aptamer, B40t77, to detect a surface glycoprotein of HIV-1, gp-120. They formed a binding assay on the glass surface that was amplified into an optical signal by LC. This high specific gp-120 detection technique had LOD at 1 µg/mL, offering potential in early stage HIV-1 detection [122].

**Figure 8:**
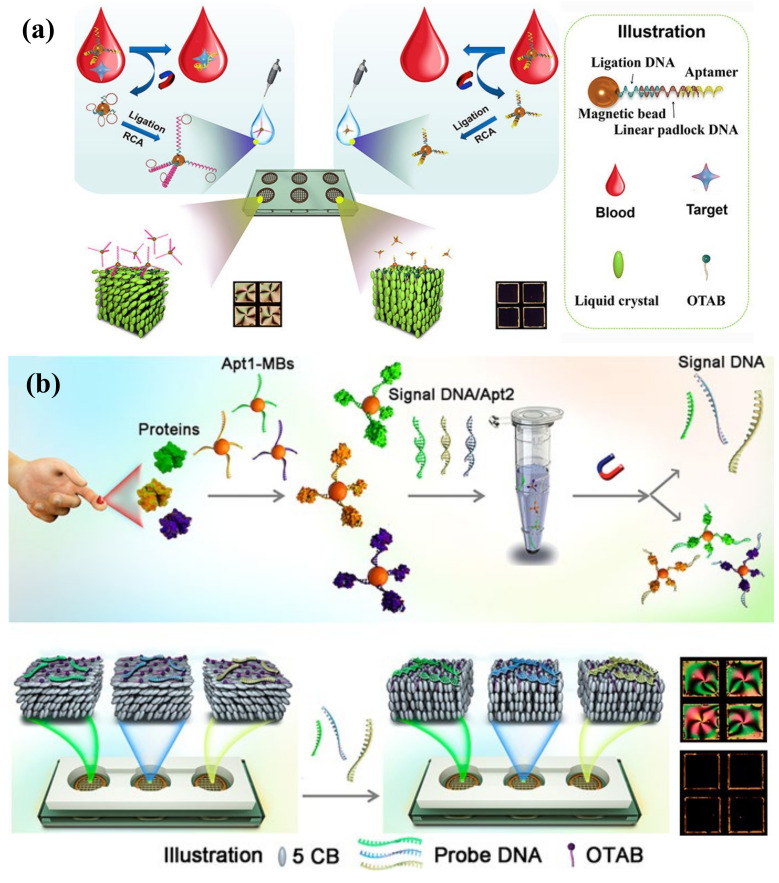
(**a**) Schematic view of LC-based biomarker sensing platform by using in situ RCA on MBs. In the presence of the target, in situ RCA is triggered to amplify the padlock DNA on MBs and LCs adopt a planar orientation at the aqueous/LC interface, inducing bright appearance of LCs. Adapted with permission from Ref. [123]. 2019, American Chemical Society. (**b**) Detection scheme of the LC-based multiplex sensor of tumor markers based on target-induced dissociation of the aptamer. Tumor markers are captured by apt1-coated MBs and trigger release of signal DNA after incubation with signal DNA/apt2 duplexes. Due to specific hybridization of signal DNA to each corresponding probe DNA, the orientation of LC changes from planar to homeotropic state, resulting in bright-to-dark appearance. Adapted with permission from Ref. [124]. 2020, American Chemical Society.

The accurate measurement of biomarkers in body fluids is critical for disease diagnosis and treatment. Taking the matrix interference effects and cross-reactivity of probe/target sets in the blood samples into consideration, the improvement in the specificity is in high demand for practical applications of LC biosensors. As shown in Figure 8a, Qi and co-workers employed in situ rolling circle amplification (RCA) on magnetic beads (MBs) triggered by aptamer–target recognition. The MBs were preassembled with ligation DNA, linear padlock DNA, and aptamers, which formed long-chain DNA strands with the presence of biomarkers. The LC–aqueous interface was decorated with OTAB at first and the RCA products were able to absorb on it to induce planar alignment of LC. Through this special method, platelet-derived growth factor BB (PDGF-BB) and adenosine were successfully detected with LODs of 0.12 pM and 31 pM, respectively [123]. Moreover, their group also proposed an LC-based multiplex sensor for tumor markers by target-induced dissociation of the aptamer (Figure 8b). Those tumor markers, such as carcinoembryonic antigen (CEA), alpha-fetoprotein (AFP), and prostate specific antigen (PSA), were captured by aptamer 1 (apt1)-coated MBs and the release of signal DNA was triggered after incubation with duplexes of signal DNA and aptamer 2 (apt2). The signal DNA subsequently changed the LC orientation from planar to homeotropic [124]. These proposed strategies provided better selectivity to the targets. More designs on MB functionalization can be carried out to measure various biomarkers in the future.

**Table 3:** LC sensors for protein detection.

LC Material	Sensing Platform	Analytes	Detection Probe	Principle	LOD	Ref.
UV-treated 5CB	LC–aqueous	Urease/Cu^2+^	CBA	NH_3_ from urease-catalyzed reaction triggered deprotonation of CBA and LC orientational change	1 nM	[99]
5CB	LC-droplet	Urease	stearic acid	Urease activity triggers the LC droplet configuration change	\	[100]
5CB	LC-SEC substrate	Urease/Cu^2+^	SEC	Enzymatic reaction of urease leads to pH change and disassembly of SEC, inducing LC reorientation	0.03 mU/mL	[101]
dodecanal-doped 5CB	LC–aqueous	Catalase	Dodecanal, carboxylic acid	Oxidation of dodecanal by hydrogen peroxide into carboxylic acid leads to planar-to-homeotropic state transition	1 nM	[103]
5CB	LC–aqueous	AchE/inhibitor	Myr	Hydrolysis of Myr by AchE, inducing LC reorientation	0.000827 U/mL, 1 fM	[104]
5CB	LC-droplet	AchE/inhibitor	Myr	Hydrolysis of Myr by AchE, inducing radial-to-bipolar change of LC droplet	\	[105]
PBA-doped 5CB	LC-droplet	pH/penicillinase	PBA	Deprotonation of PBA, inducing LC droplet LC droplet configuration change	\	[106]
dodecyl β-d-glucopyranoside d-doped 5CB	LC–aqueous	Cellulase/cysteine	dodecyl β-d-glucopyranoside d	Enzymatic hydrolysis between cellulase and surfactant, inducing LC reorientation	1 × 10^−5^ mg/mL and 82.5 μM	[107]
OTB-doped 5CB	LC–aqueous	Carboxylesterases (CES)	OTB	Enzymatic cleavage of OTB, inducing LC reorientation	18 U/L	[108]
5CB	LC–aqueous	lipase	phospholipid	Enzymatic hydrolysis of phospholipid, inducing LC reorientation	\	[109]
5CB	LC optical fiber sensor	PLA_2_	L-DLPC	Hydrolysis between PLA2 and L-DLPC reorders LC	1 nM	[110]
DOPG-decorated 5CB	LC–aqueous	Trypsin	PLL	Enzymatic reaction with PLL induces LC reorientation	1 μg/mL	[111]
5CB	LC–aqueous	Trypsin	BSA	Enzymatic cleavage of BSA disrupts LC orientation	10 ng/mL	[113]
5CB	LC–aqueous	Trypsin	CTAB-embedded gelatin	Decomposition of gelatin releases CTAB, inducing LC realignment	0.34 ng/mL	[114]
DOPG-decorated 5CB	LC–aqueous	Thrombin	PLA	Enzymatic reaction with PLA induces LC reorientation	0.25 ng/mL	[112]
5CB	LC-solid	Thrombin	Thrombin-specific aptamer	Specific interaction between thrombin and its aptamer	1 pg/mL	[118]
5CB	LC-based assay	Protease	Casein	Enzymatic cleavage of casein into peptide fragments disrupts LC orientation	10 ng/mL	[115]
5CB	LC-based assay	Protease inhibitor	Protease	Interaction between protease inhibitors and protease	\	[116]
5CB	LC-solid	Carcinoembryonic (CEA)	CEA aptamer	Specific interaction between CEA and its aptamer	0.12 pg/mL	[119]
5CB	LC-solid	Interferon-γ (IFN-γ)	IFN-γ aptamer	Specific interaction between IFN-γ and its aptamer	17 pg/mL	[120]
5CB	LC-solid	Alpha-synuclein (α-syn)	DNA aptamer	Specific interaction between α-syn and DNA aptamer, disrupting LC alignment	50 nM	[121]
5CB	LC-solid	HIV-1 surface glycoprotein	RNA aptamer B40t77	Binding event of gp-120 and B40t77, disrupting LC alignment	1 µg/mL	[122]
5CB	LC–aqueous	Cancer biomarkers (PDGF-BB/adenosine)	MBs preassembled with ligation DNA, linear padlock DNA, and aptamers	Aptamer-target recognition triggered in situ RCA on MBs, inducing LC reorientation	0.12 pM /31 pM	[123]
5CB	LC–aqueous	Tumor markers (CEA/AFP/PSA)	Apt1-coated MBs, signal DNA and Apt2	Target-induced dissociation of the aptamer and release of signal DNA, DNA hybridization induces LC reorientation	\	[124]

### 4.4. Detection of Other Biochemical Targets

As a matter of fact, LC-based sensors can also be applied for detecting many other species in addition to the abovementioned molecules, including glucose, cholic acid, amino acid, cholesterol, lipids, cells, microorganisms, and some small organic compounds. This section covers the LC-based sensing systems of these species.

#### 4.4.1. Glucose

In the biology field, glucose has a vital function as the main energy supply of living organisms and the intermediate product of metabolism. LC-based glucose sensors have been widely reported for years, most of which were constructed by means of glucose oxidase (Gox)-catalyzed oxidation of glucose, as shown in the equation below:

(5) $$\mathrm{Glucose}+\mathrm{Gox}\to \mathrm{Glucose}\;\mathrm{acid}+H_{2}O_{2}$$

**Figure 9:**
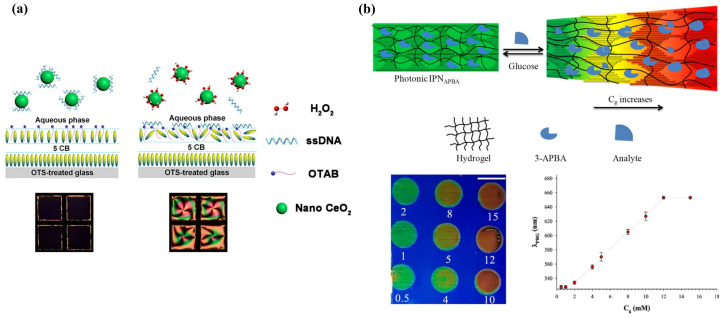
(**a**) Schematic illustration of the LC-based optical sensor for H_2_O_2_ and glucose detection. Adapted with permission from Ref. [129]. 2018, American Chemical Society. (**b**) Mechanism of the glucose detection using the IPN_APBA_ film. The binding between APBA and glucose leads to volumetric increase in the photonic IPN and reflected color change. Adapted with permission from Ref. [131]. 2019, American Chemical Society.

Glucose acid and hydrogen peroxide (H_2_O_2_) are generated after the oxidation of glucose, which, in turn, produces H^+^ and reduces pH value. Therefore, the detection of glucose was always accompanied by a change in pH value and H_2_O_2_. For enzymatic-reaction-based glucose detection, the immobilization of Gox is the primary issue. Zhong et al. prepared the Gox-modified gold grid and filled it with UV-treated 5CB, which produced 4-cyano-4′-biphenylcarboxylic acid (CBA) and became pH dependent. After immersing it in a glucose solution, the release of H^+^ led to dark-to-bright optical transition [125]. Moreover, a special pH-responsive block copolymer poly(acrylicacid-b-4-cynobiphenyl-4-oxyundecylacrylate) (PAA-b-LCP) was utilized to combine with 5CB to examine the pH change [126]. It was then studied by Kim and Khan et al. to coat the Gox-immobilized PAA-b-LCP at 5CB/aqueous interface to fabricate a glucose sensor, either in a film or droplet format. Basically, the shrinkage and swelling of PAA brushes happened with the increase in pH, leading to an ordering change in LC molecules. For a PAA-b-LCP-functionalized LC droplet, it underwent a radial-to-bipolar change in configuration at glucose concentration as low as 0.03 mM [127]. Further, with the assistance of horseradish peroxidase (HRP)-catalyzed reduction of H_2_O_2_ in Gox/HRP bienzyme system, they found that the generation of OH^−^ resulted in a more stable planar-to-homeotropic orientational change at glucose concentrations of 0.02 mM [128]. Thus, effective detection of glucose can be converted into the monitoring of H_2_O_2_ as well. Qi et al. demonstrated a real-time H_2_O_2_ detection platform through disassembly of ssDNA from nanoceria (CeO_2_) under a H_2_O_2_ environment, as shown in Figure 9a. Owing to the release of H_2_O_2_ from glucose oxidation, a low amount of glucose at 0.52 μM was detected [129].

In some cases, glucose was able to be detected through non-enzymatic methods to eliminate the enzyme denaturation and stability issues. 3-aminophenyl boronic acid (APBA) was selected as the non-enzymatic moiety that interacts with glucose by Munir et al. They first functionalized the PAA-b-LCP-coated LC droplet with APBA. The binding between APBA and glucose generated protons and decreased pH value, transferring the LC droplet from radial to bipolar. The assay had high selective and sensitive glucose detection up to 0.05 mM [130]. Another non-enzymatic system for glucose detection proposed by their group was an APBA-loaded intertwined polymer network (IPN) film, as depicted in Figure 9b. It was composed of poly(acrylic acid) (PAA) networks and CLC film. The CLC solid film was formed by UV curing of the mixture of reactive mesogens and chiral dopant, which exhibits a photonic band gap that can reflect certain wavelengths of light. After removing the unreacted chiral dopant, the remaining interspaces of CLC solid film allowed the infiltration of the functional materials. The immobilization of APBA in the PAA hydrogel network reacted with glucose, leading to the expansion of the photonic IPN and reflective color change. With the increase in glucose concentration, the reflected wavelength experienced a redshift. This photonic array sensor with LOD of 0.35 mM allowed naked-eye sensing of the glucose level in human serum [131]. Meanwhile, the CLC-hydrogel-IPN array can also be functionalized with enzymes (e.g., urease) to achieve the detection of urea [132]. The photonic IPN structure was further intertwined with CLC droplets to fabricate an optical sensor for pH, divalent metal ion, urea, and glucose detections. The CLC droplets performed as individual sensors in the dotted PAA-patterned array film with reflected color change [133].

#### 4.4.2. Cholesterol and Bile Acid

Cholesterol is a major steroid compound in mammals, which is involved in the formation of cell membranes and the synthesis of hormone and bile acids. As an important clinical index for cardiovascular disorders and liver diseases, the examination of cholesterol is meaningful. Inspired by the pH-responsive enzyme-immobilized PAA brushes to detect glucose, cholesterol sensing was also able to be achieved with co-the immobilization of cholesterol oxidase (ChO) and HRP on PAA-b-LCP. The planar-to-homeotropic orientation change of 5CB in Cu grids was triggered with the H_2_O_2_ hydrolysis and change in pH [134].

**Figure 10:**
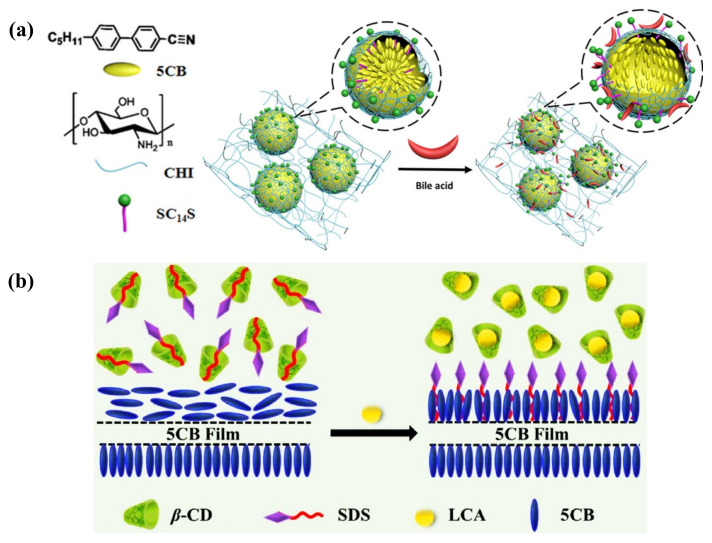
(**a**) Schematic illustration of the 5CB droplet-embedded CHI hydrogel films’ response to bile acid. Adapted with permission from Ref. [140]. 2016, American Chemical Society. (**b**) Sensing mechanism of LCA through dominant inclusion of LCA/β-CD, the expelled SDS from the cavity of β-CD re-adsorbed at the LC–aqueous interface and triggered homeotropic orientation. Adapted with permission from Ref. [142]. 2022, Elsevier.

The catabolism of cholesterol produces bile acids (BAs). It is a kind of water-soluble steroid synthesized in the liver. The most abundant primary BAs in the human body are cholic acid (CA) and chenodeoxycholic acid (CDCA). They can be transformed into secondary BAs: deoxycholic acid (DCA) and lithocholic acid (LCA), after being dehydroxylated by the intestinal bacteria [135,136]. Various forms of BAs play their physiological functions in the liver and intestine, making it necessary to detect BAs. The surfactant-laden LC–aqueous platform has been proposed to detect BA based on competitive interaction between CA and SDS at the interface [137]. A similar mechanism was also used in the LC droplet platform to detect BA, either confined in the capillary or suspended in aqueous solution, observed through optical texture change [138,139]. Even though the sensitivity was improved, it was dependent on the pH and ionic strength of the aqueous solution. Taking the stability and portability into concern, Deng et al. embedded SC_14_S-coated LC droplets in chitosan (CHI) hydrogel to obtain a hydrogel film for BA detection, which provided a more practical analytical tool (Figure 10a) [140]. In addition, to regulate the droplet size and build a more stable sensing platform, a microfluidic chip was fabricated to produce PVA/SDS-stabilized LC droplets with uniform size by Han et al. The microdroplets were trapped in the microstructure of the chip, allowing quantitative and rapid detection of BAs [141].

Ma et al. presented a strategy for LCA detection via competitive host–guest inclusion of SDS and LCA with β-cyclodextrin (β-CD), as illustrated in Figure 10b. Initially, the SDS/β-CD complex formed in the aqueous phase, keeping the LC in planar alignment. After adding LCA into the solution, it offered a dominant inclusion of LCA/β-CD. SDS was expelled from the cavity of β-CD and helped with the homeotropic alignment of LC [142]. Furthermore, this recognition probe, β-CD, was also coated on the surface of LC droplets to detect BAs. Deng et al. reported a sulfated β-CD/SC_14_S and polyelectrolyte-stabilized LC droplet system. With the adsorption of BAs at the surface, BA/SC_14_S micelles replaced the β-CD/SC_14_S complex due to the competitive host–guest recognition, leading to radial-to-bipolar configuration change. This miniaturized droplet-based sensor provided higher sensitivity and rapid detection of BAs [143]. However, a selective detection and proper classification method among different types of BAs remained an issue.

#### 4.4.3. Other Toxic Analytes

Many compounds are highly toxic, including organic vapors, pesticides, drugs, toxins, and so on. Sensing and monitoring toxic substances are of great significance in environmental protection, as well as human healthcare. As a simple and fast platform, LC-based sensors show great potential compared with the conventional expensive and complicated technologies. A brief summary of LC sensors for toxic analytes detection is shown in Table 4.

Volatile organic compounds (VOCs) are compounds with high vapor pressure and low water solubility that elicit short-term or long-term adverse health effects in humans. So far, a great number of LC-based VOC sensors has been developed to detect various chemicals, such as toluene, acetone, methanol, and butylamine. It has been suggested that most VOC sensors were designed according to the vapor-triggered LC phase transition [25]. Yet, the bulk transformation of the LC near the N-I transition point limited the practical function of the LC-based VOC sensor. Studies have been carried out on adjusting the surface topography or confined channels to encapsule LC materials to control its phase transition process and order change. Bedolla Pantoja et al. demonstrated that microwells with chemically patterned surfaces to support LC film showed an orientational response to toluene vapor. Due to the decrease in anchoring energy of LCs on the substrate under toluene vapor, the elastic energy of the strained LC film drove it into a homeotropic state [144]. The nanoparticle-patterned substrate-mediated LC film was also exploited to fabricate a VOC sensor; the solvent vapor-induced phase transition was converted into electric resistance change. Through the electrical signal, not only the phase transition of LCs, but also the type and diffusion/absorption rate of the VOCs can be determined [145]. Moreover, confined geometries, such as polymer fibers or enclosed microchannels, provided a reliable platform for LC to be used in VOC sensing. Wang et al. showed the LC/polymer fiber mats responded to toluene and acetone vapors, changing the optical transmittance of the film [146]. Liu and Zhan et al. proposed a polymer-network-stabilized liquid crystal (PNLC) system for quantitative measurement of toluene vapor concentration. The LCs were stabilized by the polymer network in the microchannel and N-I transition process was restrained. A toluene vapor-induced color profile was observed in the PNLC by the naked eye, where the colors can reflect the vapor concentration [147,148].

**Figure 11:**
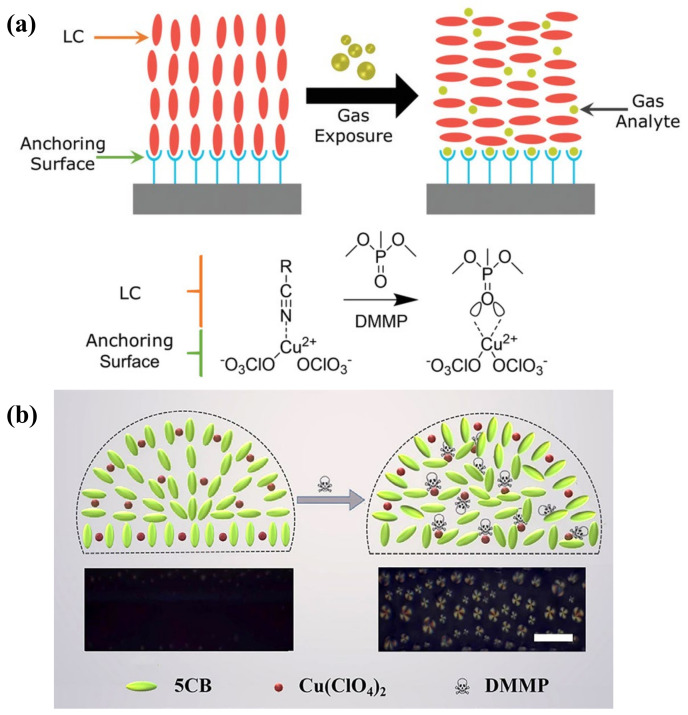
(**a**) LC orientational transition on a chemically functionalized surface upon gas exposure. The homeotropic–to–planar orientation change is triggered by the competitive binding between gas analyte and LC for the binding sites at the substrate. Adapted with permission from Ref. [156]. 2020, Wiley. (**b**) Schematic view of detection of the DMMP vapor by using Cu(ClO_4_)_2_-doped 5CB droplet. Adapted with permission from Ref. [153]. 2022, Elsevier.

Another category of organic pollutants is organophosphorus compounds, which are widely used in organophosphate nerve agents and pesticides. The studies about LC-based sensors for detecting organophosphates have been developed for years. It can be seen in Figure 11a that solid functionalized surfaces with transition metal ions were most widely utilized as binding sites for the analytes, such as copper ion (Cu^2+^) and aluminum ion (Al^3+^), with high electron affinity [149,150]. Due to the coordination interaction between the metal ions and nitrile group of cyanobiphenyl-based LCs (e.g., 5CB, E7), LC molecules perform a homeotropic orientation of LC on the surface. For the case of a dimethyl methylphosphonate (DMMP) sensor, a series of substrate modification methods was developed to support the LC film by Abbott et al. Consider, for instance, a micropillar array or microgroove-patterned amino-terminated organic substrate modified with copper perchlorate (Cu(ClO_4_)_2_), gold-coated glass substrate with a self-assembled monolayer (SAM) of 11-mercaptoundecanoic acid (MUA) modified with aluminum perchlorate (Al(ClO_4_)_3_) or mixed anions [149,150,151,152]. When DMMP with binding affinity to the metal ions was imported, the competitive interaction between LC and analytes occurred and disturbed the LC orientation. To simplify the sophisticated surface modification process, Liu et al. recently proposed a DMMP sensor based on Cu(ClO_4_)_2_-doped LC droplets (Figure 11b). The dark-to-bright optical transition of LC droplets occurred due the strong coordination interaction between DMMP and Cu(ClO_4_)_2_ at a low DMMP concentration of 2 ppb [153]. Dichlorvos (DDVP), a typical organophosphate pesticide, was also sensed through an LC droplet platform based on the alkaline phosphatase (ALP) hydrolysis of sodium monododecyl phosphate (SMP) by Zhou et al. The SMP at the interface hydrolyzed by ALP brought about a bright image of the LC droplet without the presence of DDVP. When the DDVP was introduced, it coupled with ALP and inhibited the hydrolysis of SMP. The formation of an SMP monolayer at the LC droplet surface then led to a dark appearance [154]. A similar mechanism was adopted in the sensing of AchE-inhibiting pesticides based on enzymatic events of AchE and myristoylcholine (Myr) [155]. Another organophosphorus insecticide, malathion, was detected by an LC-based aptamer sensor by Nguyen et al., with LOD at 0.465 nM. LC exhibited planar alignment under the interaction between malathion aptamer and CTAB. With the addition of malathion, the orientation transferred towards a homeotropic state due to the appearance of an aptamer–malathion complex [36].

**Figure 12:**
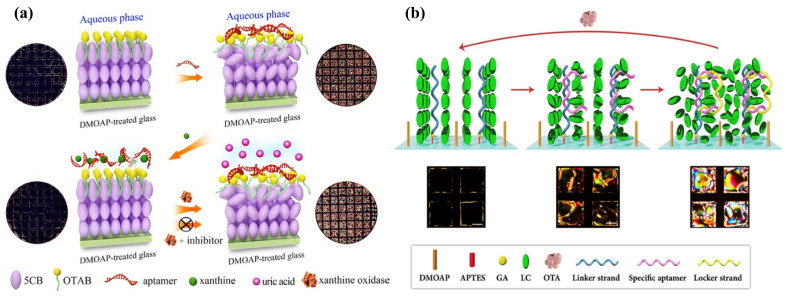
(**a**) Schematic illustration of screening the XOD inhibitors by the LC-based assay assisted with enzyme catalysis-induced aptamer release. Adapted with permission from Ref. [159]. 2021, American Chemical Society. (**b**) Diagram of the LC apta-sensing platform to determine OTA via the reorientation of LC triggered by the conformational changes of a P-shaped DNA structure, inducing a bright texture image. Adapted with permission from Ref. [165]. 2021, Elsevier.

Nowadays, many pharmaceuticals are emerging as organic chemical pollutants, including antibiotics, antibacterial drugs, painkillers, and so on. The waste produced from the pharmacy industry and drug residue may bring serious effects to the environment and human health. Therefore, it is urgent to develop sensitive and simple technologies to detect the pharmaceuticals. Here, specific aptamers were always designed and employed as sensing agents in the LC-based platform. Cocaine, as a dangerous poison, was detected with the assistance of an aptamer at the LC–aqueous interface. The aptamer presented a hairpin structure, which promoted a vertical alignment of LC. Its conformation transferred into a three-way junction structure when complexed with cocaine, inducing the planar alignment [157]. Yang et al. also proposed a DNA aptamer–surfactant-based LC sensor for ibuprofen detection. The aptamer-CTAB combination formed at the LC–aqueous surface, triggering planar orientation of LC. After introducing ibuprofen, the competitive binding of ibuprofen with the aptamer released CTAB, inducing homeotropic orientation [158]. Furthermore, Wu et al. established a special LC-based assay for screening xanthine oxidase (XOD) inhibitors, which is essential in uric-acid-lowering therapy, as shown in Figure 12a. The specific binding between xanthine and aptamer was unlocked by XOD-catalyzed oxidation, making LC transfer from a homeotropic to a planar state. On the contrary, when potent inhibitors acted on XOD, the xanthine–aptamer complex and the LC orientation maintained its original state. As a result, three effective XOD inhibitors were selected through this approach [159].

Antibiotics are chemical substances produced by microorganisms and usually used in treating infections caused by pathogens, though they still have side effects and lead to certain damage to human bodies. Research about LC-based biosensors for detecting antibiotics involves amoxicillin (AMX), kanamycin (Kana), penicillin G, etc. Nguyen et al. fabricated an LC-solid platform with an aptamer immobilized on the substrate for AMX detection. The aptamer specifically recognized the AMX and disturbed the LC orientation to generate a bright optical image [160]. Wang et al. also proposed a AuNP-enhanced LC-solid sensing method for Kana. By premixing AuNP with Kana, the interaction between immobilized aptamer and Kana was amplified through the optical signal of LC and the LOD was reduced to 0.1 pM [161]. It is more sensitive than the previous LC-droplet-based Kana sensor with an LOD of 0.17 nM [162]. Recently, a sessile LC droplet array prepared via inkjet printing was developed by Xie and co-authors to detect penicillin G. It was achieved through the penicillinase-catalyzed enzymatic hydrolysis of penicillin G; the release of H^+^, in turn, protonated PBA and led to the formation of a PBA monolayer. The molecular orientation change was visualized through axial-to-windmill texture transition under POM within several minutes, with an LOD of 0.178 ng/mL [163]. The stable polymer film for LC droplet anchoring makes it possible to fabricate a wearable sensing device, which can be applied in more fields.

As for toxins, for instance, ochratoxin A (OTA), this was regarded as a vital analyte. Khoshbin et al. presented several novel apta-sensing platforms for ultra-sensitive detection of OTA. One was based on the conformational switch of the immobilized π-shaped structure of aptamer and its complementary strands, the off–on response was initiated by the binding of OTA with an aptamer and expansion of DNA strands and observed through dark-to-bright optical change [164]. Another approach utilized three oligonucleotides to form a P-shaped structure: a linker strand, a specific aptamer, and a locker strand. As depicted in Figure 12b, the binding between OTA and aptamer disassembled the aptamer–locker hybrid, followed by disassembly of the P-shaped structure and realignment of LC. The off–on response was observed through bright-to-dark optical change [165]. Very recently, a unique LC microarray sensor for detection of aflatoxin B1 (AFB1) was demonstrated by Wang et al. The LC microarray was fabricated through LC infiltrating into the inverse opal LC polymer (IO-LCP) film with microporous structure. LC was confined in the holes in a disordered state. Due to the binding between aptamer and AFB1, complementary ssDNA was released and the degree of orientation of LC increased. This order change can be transformed into micro-spectral optical sensing signals, providing quantitative measurement of AFB1 [166].

**Table 4:** LC sensors for detection of toxic analytes.

LC Material	Sensing Platform	Analytes	Detection Probe	Principle	LOD	Ref.
5CB	LC–aqueous	Cholesterol	ChO and HRP	Enzymatic reaction of cholesterol induced pH change and LC orientational change	0.8 mM	[134]
5CB	LC–aqueous	Cholic acid	SDS	Competitive interaction between CA and SDS	10 μM	[137]
5CB	LC droplet	Cholic acid	SDS	Competitive interaction between CA and SDS	5 μM	[138]
5CB	LC droplet-based capillary platform	Bile acid	SDS	Competitive interaction between CA and SDS	\	[139]
5CB	LC droplet-embedded hydrogel film	Bile acid	SC_14_S	Competitive adsorption of the bile acid	\	[140]
5CB	LC droplet	Bile acid	SDS	Removal of SDS from the surface of LC droplet	\	[141]
5CB	LC–aqueous	Lithocholic acid	β-CD	Competitive host-guest inclusion between SDS/β-CD and LCA/β-CD complex	2 μM	[142]
5CB	LC droplet	Bile acid	β-CD	Competitive host-guest recognition induced SC_14_S displacement from β-CD	\	[143]
5CB	LC droplet	DMMP	Cu(ClO_4_)_2_	Competitive coordination interaction of Cu(ClO_4_)_2_ with DMMP and LC	2 ppb	[153]
5CB	LC droplet	DDVP	Alkaline phosphatase (ALP)	ALP hydrolysis of SMP	0.1 ng/mL	[154]
5CB	LC droplet	AChE-inhibiting pesticides	AChE	Enzymatic event of AChE and myristoylcholine (Myr)	\	[155]
5CB	LC–aqueous	malathion	DNA aptamer	Formation of aptamer-malathion complex changed LC orientation	0.465 nM	[36]
5CB	LC–aqueous	Cocaine	Aptamer	Specific interaction between aptamer and cocaine, inducing LC reorientation	1 nM	[157]
5CB	LC–aqueous	Ibuprofen	DNA aptamer	Specific binding of ibuprofen with aptamer releases CTAB, inducing LC reorientation	12.5 μg/mL	[158]
5CB	LC–aqueous	Xanthine oxidase (XOD) inhibitor	XOD and its aptamer	Xanthine oxidation by XOD prevents the specific binding of xanthine and aptamer, inducing LC reorientation	\	[159]
5CB	LC-solid	amoxicillin (AMX)	AMX aptamer	Specific interaction between aptamer and AMX, inducing LC reorientation	3.5 nM	[160]
5CB	LC-solid	Kanamycin (Kana)	Kana aptamer	Formation of AuNPs–Kana–aptamer complex induces LC reorientation	0.1 pM	[161]
5CB	LC droplet	Kanamycin (Kana)	Kana aptamer	Specific recognition of kanamycin and aptamer releases CTAB, inducing LC reorientation	0.17 nM	[162]
5CB	LC droplet	penicillin G	Penicillinase and PBA	Enzymatic hydrolysis catalyzed by penicillinase leads to PBA protonation, inducing LC reorientation	0.178 ng/mL	[163]
5CB	LC-solid	ochratoxin A (OTA)	π-shaped aptamer	Conformational switch of aptamer when bind with OTA, inducing LC reorientation	0.63 aM	[164]
5CB	LC-solid	ochratoxin A (OTA)	P-shaped structure of DNA strands	Binding between OTA and aptamer disassembles the aptamer-locker hybrid, inducing LC realignment	0.0078 aM	[165]
5CB	LC microarray	Aflatoxin B1 (AFB1)	ssDNA	Binding between aptamer and AFB1 release complementary ssDNA, inducing LC order change reported by micro-spectral optical signal	300 pM	[166]

## 5. Machine-Learning-Assisted LC Sensor

According to the discussion above, the LC-based sensor has been demonstrated as a promising tool in the biochemical analytical field. The molecular signal was generally reported through the change in LC orientation, then amplified and transduced into optical signal through POM. The concentration of the analyte is usually related to the average gray value of the optical image, which can be calculated through the software Image J or Adobe photoshop. However, the current technology relying on POM has a few limitations: (1) expensive and bulky POM microscopes accompanied by a computer are required to capture optical images; (2) the efficiency and accuracy of the naked-eye analysis of a large number of optical images is hard to achieve; and (3) it is quite hard to distinguish the responses of the LC film or droplet to some analytes (e.g., vapor-phase analytes). For the purpose of enhancing the accuracy, selectivity, and automaticity of the LC-based sensing platform, as well as simplifying and miniaturizing the sensing device, artificial intelligence and machine learning are becoming an emerging auxiliary tool in developing LC-based biochemical intelligent sensors.

**Figure 13:**
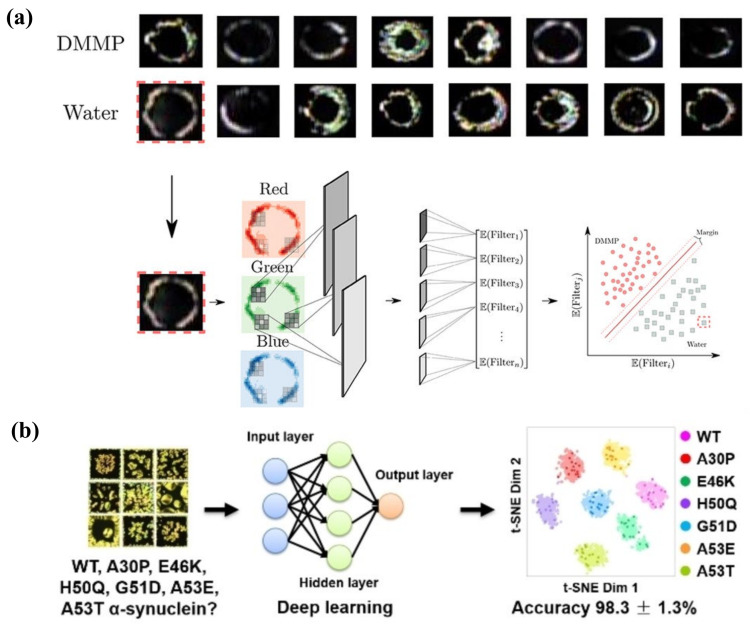
(**a**) Optical responses of LCs under N_2_–water (30% relative humidity) and N_2_–DMMP (10 ppm) environments. Feature extraction and classification framework with CNN VGG16. Adapted with permission from Ref. [167]. 2020, American Chemical Society. (**b**) Identifying wild-type and six familial mutant α-synucleins through the deep learning and LC biosensor combination system. Adapted with permission from Ref. [168]. 2021, Wiley.

Smith et al. demonstrated a machine learning (ML) algorithm to improve the selectivity and sensing speed of the LC-based gas sensor, as shown in Figure 13a. Advanced techniques were employed to extract the feature information from grayscale images of LC triggered by different gas analytes. Next, the information was exploited in the classification model, AlexNet, to train accurate classifiers. This framework showed over 99% accuracy in classifying thousands of optical images under the presence of DMMP vapor or nitrogen stream, with 30% relative humidity (RH). Furthermore, this group extended the study to VGG16, a convolutional neural network (CNN) embedded with a more compact set of convolutional filters than AlexNet, to extract features from colored images. A great reduction in feature number was achieved, with minimal losses in accuracy. It also revealed that the hue distributions offered useful information for image characterization [167].

The practical application of machine learning in LC sensing also involved disease-related biomolecule analysis. Xu et al. designed an LC-based diagnostic kit for ultrasensitive detection of SARS-CoV-2 ssRNA. It was then upgraded into a smartphone-based application (app) using a machine learning strategy, leading to great potential in self-testing for SARS-CoV-2 [72]. Yang et al. developed a phospholipid-decorated LC–aqueous system to explore α-synuclein and phospholipid interaction, which is essential in the pathogenesis and diagnosis of Parkinson’s disease. The overall system implementation is shown in Figure 13b. Through the combination of deep learning, the distinct features formed in LC images by wild-type and six familial mutant α-synucleins were recognized with a high accuracy of 98.3 ± 1.3%. It provided a reliable platform for α-synuclein mutation identification in clinical applications [168].

As for LC droplets, it is difficult to identify the internal orientation precisely via optical texture. With the assistance of CNN, Frazão et al. presented an LC droplet recognition system to analyze the optical texture dynamics to various VOCs. It not only successfully classified 11 VOCs of distinct structures, but also predicted the concentration of acetone vapor exposed to the system [169]. However, it only reported the phase-change-induced optical texture change in droplets; the orientational change induced radial-to-bipolar change was not detected through machine learning. Based on the analysis of the internal configurations of LC droplets using flow cytometry, Jiang et al. applied the CNN Endonet to analyze the FSC (forward scattering)/SSC (side scattering) scatter plots to predict the internal configuration of LC droplets [170]. Hence, the source and concentration of endotoxin were predicted indirectly through the scatter field and Endonet, which induced a bipolar-to-radial transition of LC droplets [171].

Therefore, the combination of an LC-based sensor and machine learning provides great potential in the future development of biochemical sensing platforms. It will speed up the sensing process and pave the way for the production of the miniaturized, integrated, and commercially available detectors for various analytes. It is highly expected that more image processing, feature extraction, and classification algorithms should be studied to realize precise target recognition.

## 6. Conclusions and Future Perspectives

This paper reviewed most of the state-of-the-art research about LC-based sensors in the biochemical field. The ultrasensitive LC-based sensing platform is attributed to the unique physical characteristics of LC, possessing a rapid response to various external stimuli and signal amplification ability. The combination of LC and functional molecules offers multiple features and benefits to develop selective, simple, and label-free LC-based sensors for detecting many types of biomolecules or chemical analytes. Up to now, great achievements have been made in LC-based biochemical sensors, thanks to the efforts of researchers around the world. However, they still face some challenges in practical applications:

(1) Many LC sensors are non-reproducible, since some reactions between targets and probes are irreversible, such as enzyme hydrolysis and DNAzyme cleavage. Fabricating a reusable LC sensing platform is good for waste and cost reduction.
(2) The stability of the LC-based platforms should be taken into consideration, especially LC–aqueous and LC droplet systems, since they are easily influenced by the environmental conditions (e.g., temperature, light, mechanical disturbance).
(3) Some LC-based sensors can only achieve qualitative detection of the analytes, especially for LC droplet sensors; it is hard to distinguish tiny differences in the optical texture during the pre-radial state. How to achieve accurate quantitative analysis and standardize the parameters are essential questions in the LC sensing field.
(4) Even though the LC sensor exhibits good sensitivity, it still faces certain gaps with some conventional sensors. The LOD needs further improvement by developing more signal amplification strategies.
(5) Currently, the most commonly used material in LC sensors is nematic LC 5CB or E7. More LC materials should be explored in LC-based sensors, such as 8CB, 7CB, and cholesteric LC. Their special properties might provide new thoughts and breakthrough in LC sensors.
(6) In fact, POM is always the most effective tool to report the LC orientation change. Besides the optical method, more signal transduction methods should be explored in the LC sensing platform, such as spectral, electronic, dielectric, fluorescence signal, and so on.
(7) As mentioned in Section 5, despite a few works reporting the utilization of machine learning in LC-based sensors, this field is still in a fledging period. Therefore, more efforts are required to enhance artificial intelligence technology and it is expected to see breakthroughs.

The current development in LC sensors is mainly at the stage of laboratory research. This label-free, fast, simple, and cost-effective strategy shows great superiority compared with other complicated conventional techniques. With the increased demand for point-of-care testing and on-site detection in the clinical field, as well as home-based self-testing by consumers, the future goal of LC-based sensors is commercialization. By accompanying machine learning and artificial intelligence technology, we are looking forward to seeing the LC-based biochemical sensor developing into a portable, convenient, intelligent, and user-friendly device in the near future.

## Figures and Tables

**Table 2:** LC sensors for nucleic acids detection.

LC Material	Sensing Platform	Analytes	Detection Probe	Principle	LOD	Ref.
**5CB**	LC-solid	DNA target strand	ssDNA probe	DNA hybridization induces LC orientational change	\	[61,62,63,64]
**5CB**	LC-solid	DNA	PNA (Na^+^)-doped LC	Complexation between negatively charged DNA and metal ions	10 fM	[65]
**5CB**	LC-solid	DNA target	ssDNA probe	DNA hybridization induced increase in surface coverage	0.1 nM	[66]
**5CB**	LC–aqueous	ssDNA/dsDNA	Surfactant	ssDNA-surfactant complex induces LC reorientation	\	[69,70]
**5CB**	LC–aqueous	p53 mutation gene segment	DNA dendrimer	Formation of target-triggering DNA dendrimers induces LC reorientation	0.08 nM	[71]
**5CB**	LC–aqueous	Pathogen genomic DNAs	ssDNA probe/DTAB	Absorption of ssDNA targets with probes induces LC vertical alignment by DTAB	0.05 nM	[34]
**E7**	LC–aqueous	SARS-CoV-2 ssRNA	Complementary ssDNA probe/ DTAB	Absorption of ssRNA targets with probes induces LC vertical alignment by DTAB	30 fM	[72]
**5CB**	LC–aqueous	complementary DNA targets	cholesterol-labeled DNA probes	Hybridization of self-assembled of cholesterol-labeled DNA probes with targets induces LC reorientation	51 μg/ml	[73]
**5CB**	LC–aqueous	complementary DNA targets	DNA–lipids	DNA hybridization causes de-assembly of DNA–lipids, inducing optical image change	\	[74]
**5CB**	LC droplet	ssDNA/dsDNA	PLL	Electrostatic interaction between ssDNA and PLL induces LC droplets configuration change	\	[75]

## Data Availability

Not applicable.
